# N-acetylcysteine use among patients undergoing cardiac surgery: A systematic review and meta-analysis of randomized trials

**DOI:** 10.1371/journal.pone.0213862

**Published:** 2019-05-09

**Authors:** José Eduardo G. Pereira, Regina El Dib, Leandro G. Braz, Janaina Escudero, Jason Hayes, Bradley C. Johnston

**Affiliations:** 1 Department of Anesthesiology, Botucatu Medical School, Universidade Estadual Paulista, Botucatu, São Paulo, São Paulo, Brazil; 2 Department of Anesthesiology, EsSEx, Hospital Central do Exército, Rio de Janeiro, Rio de Janeiro, Brazil; 3 Department of Anesthesiology, Santa Casa de Misericórdia de Barra Mansa, Barra Mansa, Rio de Janeiro, Rio de Janeiro, Brazil; 4 Department of Community Health and Epidemiology, Dalhousie University, Faculty of Medicine, Halifax, Canada; 5 Institute of Science and Technology, Univ Estadual Paulista, São Paulo, São José dos Campos, Brazil; 6 McMaster Institute of Urology, McMaster University, Hamilton, Ontario, Canada; 7 Department of Anesthesia and Pain Medicine, The Hospital for Sick Children, University of Toronto, Toronto, Ontario, Canada; University of British Columbia, CANADA

## Abstract

**Background:**

Cardiac surgeries are complex procedures aiming to re-establish coronary flow and correct valvular defects. Oxidative stress, caused by inflammation and ischemia-reperfusion injury, is associated with these procedures, increasing the risk of adverse outcomes. N-acetylcysteine (NAC) acts as an antioxidant by replenishing the glutathione stores, and emerging evidence suggests that NAC may reduce the risk of adverse perioperative outcomes. We conducted a systematic review and meta-analysis to investigate the addition of NAC to a standard of care among adult patients undergoing cardiac surgery.

**Methods:**

We searched four databases (PubMed, EMBASE, CENTRAL, LILACS) from inception to October 2018 and the grey literaure for randomized controlled trials (RCTs) investigating the effect of NAC on pre-defined outcomes including mortality, acute renal insufficiency (ARI), acute cardiac insufficiency (ACI), hospital length of stay (HLoS), intensive care unit length of stay (ICULoS), arrhythmia and acute myocardial infarction (AMI). Reviewers independently screened potentially eligible articles, extracted data and assessed the risk of bias among eligible articles. We used the GRADE approach to rate the overall certainty of evidence for each outcome.

**Results:**

Twenty-nine RCTs including 2,486 participants proved eligible. Low to moderate certainty evidence demonstrated that the addition of NAC resulted in a non-statistically significant reduction in mortality (Risk Ratio (RR) 0.71; 95% Confidence Interval (CI) 0.40 to 1.25), ARI (RR 0.92; 95% CI 0.79 to 1.09), ACI (RR 0.77; 95% CI 0.44 to 1.38), HLoS (Mean Difference (MD) 0.21; 95% CI -0.64 to 0.23), ICULoS (MD -0.04; 95% CI -0.29 to 0.20), arrhythmia (RR 0.79; 95% CI 0.52 to 1.20), and AMI (RR 0.84; 95% CI 0.48 to 1.48).

**Limitations:**

Among eligible trials, we observed heterogeneity in the population and interventions including patients with and without kidney dysfunction and interventions that differed in route of administration, dosage, and duration of treatment. This observed heterogeneity was not explained by our subgroup analyses.

**Conclusions:**

The addition of NAC during cardiac surgery did not result in a statistically significant reduction in clinical outcomes. A large randomized placebo-controlled multi-centre trial is needed to determine whether NAC reduces mortality.

**Registration:**

PROSPERO CRD42018091191.

## Introduction

Coronary artery disease is the most common cardiac disease worldwide with approximately 500,000 new and 300,000 recurrent events each year in the United States alone [1]. Almost 300,000 patients were submitted to cardiac surgeries in the United States according to the executive summary of the Society of Thoracic Surgeons in 2016 [2], and in Brazil, 21,474 coronary artery bypass graft and 6,803 heart valve replacement surgical procedures were performed in 2017 [3].

Cardiac surgery is commonly performed on-pump, which includes the assistance of cardiopulmonary bypass (CPB), where a device substitutes the heart and lungs to pump and oxygenate circulating blood. However, a considerable number of coronary artery surgical procedures are performed off-pump without CPB assistance [4–6]. Cardiac surgery aims to correct valvular defects, and re-establish coronary blood flow, relieving angina and dyspnea symptoms, but is accompanied by a risk of complications that might affect organs such as the lung [7,8], kidney [9–11], brain [12–15] and even the heart [16–18].

Complementary or co-administered therapies are commonly considered in perioperative medicine. For instance, agents with antioxidant properties such as n-acetylcysteine (NAC) may reduce oxidative stress [19–20] and inflammation [21–25] among patients undergoing cardiac surgery and potentially reduce postoperative complications.

Previous reviews [26–31] have assessed NAC administration during cardiac surgery. Reviews to date are, however, limited in that they do not include all studies assessing the most patient-important outcomes such as arrhythmia [32], mortality [33] and hospital length of stay [33, 34] [26, 28, 29, 31], they have not conducted a comprehensive literature search [26–30] and they have only considered publications in English [26]. In addition, they have excluded randomized controlled trials (RCTs) that administered NAC through cardioplegia solution [27], and they did not use the Grading of Recommendations Assessment, and the Development and Evaluation (GRADE) approach to rate the certainty of evidence.

We, therefore, conducted a more comprehensive systematic review of RCTs to assess whether the perioperative addition of NAC to the standard treatment of adult patients submitted to cardiac surgeries reduces mortality as well as secondary outcomes (i.e. acute renal insufficiency, cardiac insufficiency, hospital and/or intensive care unit length of stay, adverse postoperative outcomes) when compared to standard of care alone.

## Methods

The Cochrane Handbook for Intervention Reviews [35] guided our choice of methods. This review was registered with PROSPERO (International Prospective Register of Systematic Reviews) under the number CRD42018091191. We report our results following the PRISMA (Preferred Reposting Items for Systematic Reviews and Meta-analysis) statement [36].

### Eligibility criteria

We considered all RCTs evaluating n-acetylcysteine (NAC) compared to standard of care (SoC) plus placebo, or SoC alone, in adults’ patients (aged 18 years and above) undergoing on-pump or off-pump cardiac surgery.

The primary outcome of this review was mortality, the most patient important endpoint. Secondary outcomes were the following: acute renal insufficiency; cardiac insufficiency; hospital and/or intensive care unit length of stay and adverse postoperative outcomes (e.g., arrhythmia, AMI).

Eligible studies reported on one or more of the outcomes are listed above.

### Data source and searches

The search was performed in the following electronic databases: the Cochrane Central Register of Controlled Trials (CENTRAL, 2018), PubMed (OvidSP, 1966 to 2018), EMBASE (Excerpta Medica database) (OvidSP, 1980 to 2018) and LILACS (Literatura Latino-Americana e do Caribe em Ciências da Saúde) (1982 to 2018). The databases were searched for published RCTs in humans, from inception to 10^th^ October 2018. No restrictions were placed on language or publication status.

The search was conducted using multiple combinations of the following keywords: “coronary artery disease”, “cardiac surgery”, and “n-acetylcysteine” (S1 Table).

In addition, an online hand-searching for additional eligible studies was conducted in the search engine of three major anesthesiology journals (Anesthesia and Analgesia; Anesthesiology; European Journal of Anaesthesiology) from inception to June 2018 (S2 Table), and we also searched the reference lists of potentially eligible studies, conferences proceedings, previous existing systematic reviews, and we searched the clinicaltrials.com registry.

#### Selection of studies

Using standardized screening forms, two reviewers (JEGP, RED) independently screened all titles and abstracts identified by the literature search, obtained full-text articles of all potentially eligible studies, and evaluated these studies for eligibility. Reviewers resolved the disagreement through discussion, and with third-party adjudication if necessary.

### Data extraction and risk of bias assessment

Two reviewers (JEGP, RED) independently extracted the following data using a pre-piloted, standardized data extraction form (S3 Table): characteristics of the study design; participants; interventions; outcomes and the length of follow-up. If eligible articles had missing data we contacted authors for clarification.

Reviewers independently assessed the validity of included studies using the risk of bias approach for Cochrane reviews of RCTs as modified by Busse and Guyatt [37,38]. Risk of bias was assessed using five separate criteria: adequacy of sequence generation, allocation sequence concealment, blinding (investigators, patients, collectors, statistician, and outcome assessors), incomplete outcome data, and selective outcome reporting. For incomplete outcome data, we considered loss to follow-up of less than 10%, and a difference of 5% or less in missing outcome data between intervention and control groups as low risk of bias.

### Certainty of evidence

We used the Grading of Recommendations Assessment, Development and Evaluation (GRADE) approach to rate the certainty of evidence, in which a body of evidence based on randomized trials begins as high certainty evidence but may be rated down by one or more levels for each of five categories of limitations: risk of bias, inconsistency, indirectness, imprecision and reporting bias [38]. Detailed GRADE guidance was used to assess the overall risk of bias [39], imprecision [40], inconsistency [41], indirectness [42] and publication bias [43], and results were summarized in an evidence profile table.

### Data synthesis and statistical analysis

We calculated pooled risk ratios (RRs) for dichotomous outcomes and mean differences (MDs) for continuous outcomes, with the corresponding 95% confidence interval (CI). We used a random-effects model with the Mantel-Haenszel statistical method for the dichotomous outcomes and the Inverse Variance for the continuous outcomes. We addressed variability in results across studies using the I^2^ statistic and the P value (> 0.10) obtained from the Cochrane chi-square test.

Risk ratio does not incorporate zero-event trials, thereby excluding these trials and data from the combined estimate. A random-effect model was chosen because when dealing with a series of studies, subjects typically differ substantially from one study to another [44].

Our primary analyses were based on all randomized patients who had reported outcomes for each study (complete case analysis). We used Review Manager (RevMan) (version 5.3; Nordic Cochrane Centre, Cochrane) for all analyses [45].

We performed pre-specified subgroup analyses, stratifying by route of administration of NAC (intravenous, cardioplegia, oral, oral plus intravenous) [46,47]; NAC dose (< 100, 100 to < 300, ≥ 300 mg.kg-1.day-1) [47]; duration of NAC (< 24, 24–48, > 48 hours) [48]; surgical technique (CPB, no CPB) [49]; anesthesia technique (inhalational, total intravenous) [49], and patient characteristics (i.e., kidney dysfunction, limited cardiac ejection fraction). Further, among subgroup effects demonstrating a significant test of interaction, we assessed the credibility of the observed effect using published assessment criteria including whether: 1) there was a low likelihood that chance explains the observed effect; 2) the effect was consistent across studies; 3) the subgroup hypothesis was specified a priori with the direction of the subgroup effect specified a priori; 4) there was strong existent biological support (biological rationale); and 5) the evidence was supporting the effect based on within–or between study comparisons [50].

We assessed for publication bias using visual inspection of funnel plots for outcomes with 10 or more studies [51].

## Results

### Search results

**Fig 1:**
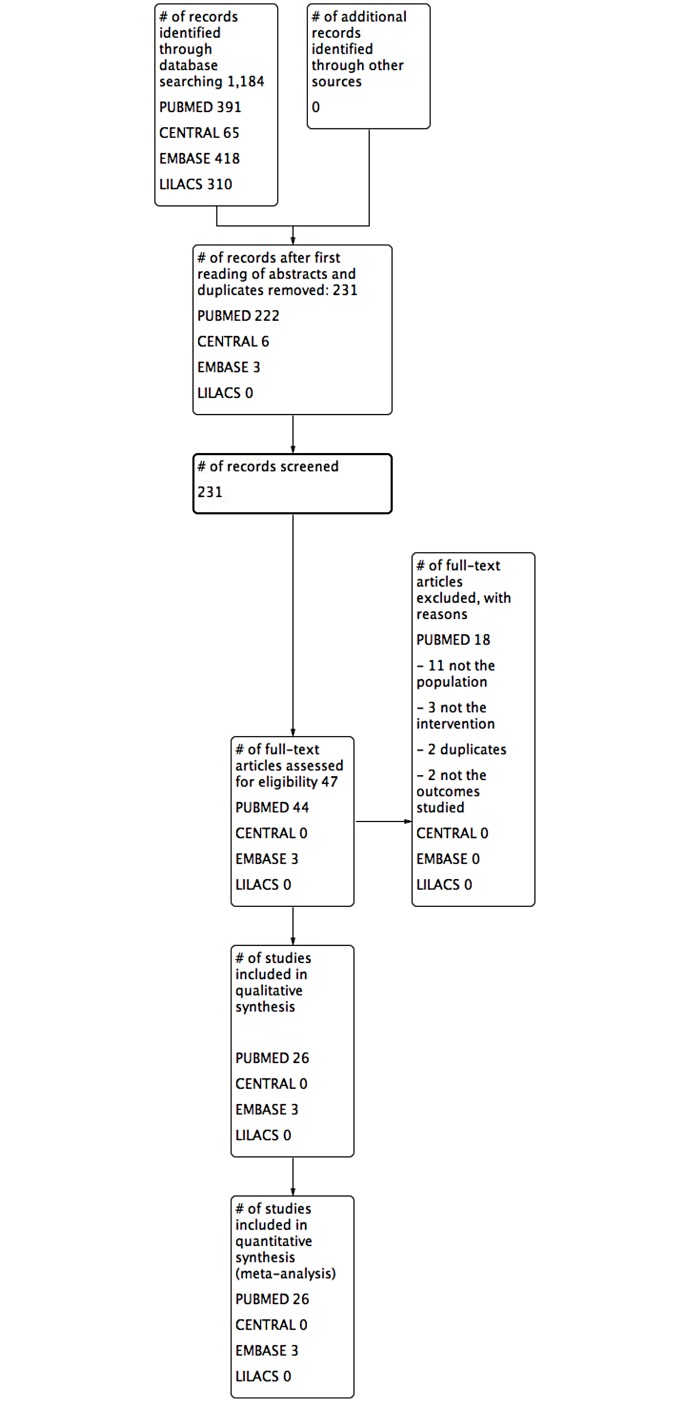
PRISMA flowchart.

We identified a total of 1,189 citations and after independent screening by title, and then by abstract, we obtained full-text copies for 47 citations that were potentially eligible for inclusion in the review. Of those, 18 did not fulfill our eligibility criteria and were excluded (S4 Table). We, therefore, included 29 studies [20, 52–79] with a total of 2,486 participants in this review (Fig 1). No additional eligible studies were identified based on hand-searching of anesthesiology journals for relevant primary studies.

### Characteristics of the included studies

Among the 29 eligible RCTs, trials took place in a variety of settings (S5 Table) including Turkey [20, 53, 54, 59, 60, 63, 66, 67, 69, 70], Canada [56, 58, 79], United States of America [52, 55], Finland [73, 78], Germany [61], Italy [75], Belgium [57], India [68, 71, 72], South Korea [65, 77], Iran [64, 76], Brazil [74] and Australia [62]. Sample sizes ranged from 18 [57] to 295 [56] participants (S5 Table).

A total of 21 trials including 1,468 patients were treated under CPB, while eight trials involving 1,018 patients were treated off-pump (S6 Table). According to the anesthetic technique, six trials [20, 59, 66, 69, 76, 78] used total intravenous anesthesia technique (TIVA), with a total of 348 participants, while 15 trials [54, 57, 60, 63, 65, 67, 68, 70–75, 77, 79] reported using inhaled anesthetics with a total of 1,203 participants. Eight trials [52, 53, 55, 56, 58, 61, 62, 64] with a total of 936 participants did not report which anesthetic technique was employed (S6 Table).

All except one of our eligible trials included both male and female participants, with one trial [52] including only male patients. The mean age of the participants in the NAC group ranged from 54 years to 74 years, with a mean age of 64 years for the NAC group. The mean age of the participants in the control group ranged from 53 years to 73 years, with a mean age for the control group of 66 years (S5 Table).

Ten RCTs with a total of 1,261 participants [52, 55, 56, 62, 72–75, 77, 79] included patients with kidney dysfunction only. Four trials with a total of 530 participants [56, 65, 72, 77] included only patients with low cardiac ejection fraction (< 0.4) (S5 Table).

### Risk of bias in individual studies

Allocation concealment was a significant limitation in 8 trials and judged to be at high risk of bias [20, 59, 61–64, 68, 74]. Blinding of participants was judged to be at high risk of bias in one trial [68], while blinding of both personnel and outcome assessors were considered at high risk of bias in 8 trials [20, 63, 65–68, 72, 78]. Two trials [77, 79] were considered high risk for selective reporting, one [79] for not reporting creatinine clearance results, and the other [77] for not reporting mortality results under their respective protocols. Incomplete outcome data was considered at high risk of bias in one trial [55] due to total loss to follow-up of 10.25% and in another trial [52] due to a between-group difference in loss to follow-up of 5.5% (S1 Fig and S7 Table).

### Effectiveness of interventions

#### Mortality

**Fig 2:**
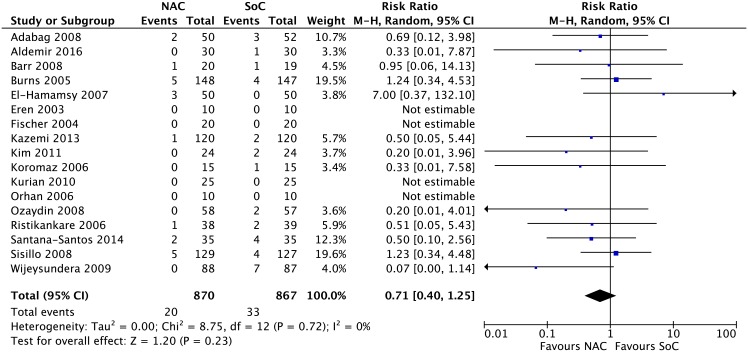
Meta-analysis on mortality.

Results from 17 RCTs [52, 53, 55, 56, 58, 60, 61, 64, 65, 67–70, 73–75, 79] including 1,737 patients yielded a non-statistically significant difference between NAC and SoC on the reduction of mortality (RR 0.71, 95% CI 0.40 to 1.25; events (NAC:20/870, SoC:33/867); I^2^ = 0%; p = 0.23) (Fig 2). The certainty of evidence was rated as low because of imprecision (low number of events (<400) and wide confidence intervals including clinically important benefit and harm) (Table 1) and no publication bias was detected (S2 Fig, panel A).

**Table 1:** GRADE evidence profile for clinical outcomes.

Quality assessment						Summary of findings					Certainty in estimates
						Study event rates		Relative risk or average (CI 95%)	Anticipated absolute effects		
No of participants (studies) Follow-up in days	Risk of bias	Inconsistency	Indirectness	Imprecision	Publication bias	Control	NAC		Control^1^	NAC	
**Mortality**											
1,737 (17) 2–90 days	No serious limitations	No serious limitations	No serious limitations	Very serious imprecision^3^	Undetected	33/ 867	20/870	**0.71** (0.40–1.25)	27 per 1000	8 fewer per 1000 (16 fewer to 7 more)	⨁⨁ΟΟ LOW
**Acute Renal Insufficiency**											
1,711 (15) 1–60 days	No serious limitations	No serious limitations	No serious limitations	Very serious imprecision^3^	Undetected	193/ 845	180/866	**0.92** (0.79–1.09)	511 per 1000	40 fewer per 1000 (107 fewer to 46 more)	⨁⨁ΟΟ LOW
**Cardiac Insufficiency**											
1,149 (12) 2–60 days	No serious limitations	No serious limitations	No serious limitations	Very serious imprecision^3^	Undetected	24/577	19/572	**0.77** (0.44–1.38)	83 per 1000	19 fewer per 1000 (46 fewer to 31 more)	⨁⨁ΟΟ LOW
**Hospital length of stay**											
1,650 (18) 2–60	No serious limitations	Serious limitations^2^	No serious limitations	No serious imprecision	Undetected				Mean HLoS with NAC was -0.21 days	Average 0.21 fewer days (0.64 fewer to 0.23 more)	⨁⨁⨁Ο MODERATE
**Intensive care unit length of stay**											
1,512 (17) 2–60	No serious limitations	Serious limitations^2^	No serious limitations	No serious imprecision	Undetected				Mean ICULoS with NAC was -0.04 days	Average 0.04 fewer days (0.29 fewer to 0.20 more)	⨁⨁⨁Ο MODERATE
**Arrhythmia**											
886 (10) 2–60 days	No serious limitations	No serious limitations	No serious limitations	Very serious imprecision^3^	Undetected	98/ 440	81/446	**0.79** (0.52–1.20)	460 per 1000	96 fewer per 1000 (220 fewer to 92 more)	⨁⨁ΟΟ LOW
**Acute Myocardial Infarction**											
1178 (11) 1–15 days	No serious limitations	No serious limitations	No serious limitations	Very serious imprecision^3^	Undetected	26/591	22/587	**0.84** (0.48–1.48)	92 per 1000	15 fewer per 1000 (48 fewer to 44 more	⨁⨁ΟΟ LOW

HLoS: hospital length of stay; ICULoS: intensive care unit length of stay; NAC: n-acetylcysteine.

^1^Baseline risk estimates come from control arm of the greater weight randomized trial in the meta-analysis.

^2^There was serious limitation related to inconsistency (I^2^ > 50%).

^3^There was very serious limitation related to imprecision (rated down twice due to low number of events and wide confidence intervals including clinically important benefit and harm).

With respect to subgroups of interest, we found no statistically significant differences based on route, dose, timing of administration of NAC, surgical or anesthetic technique and characteristics of the population (S3 Fig, panel A).

#### Secondary outcomes

**Fig 3:**
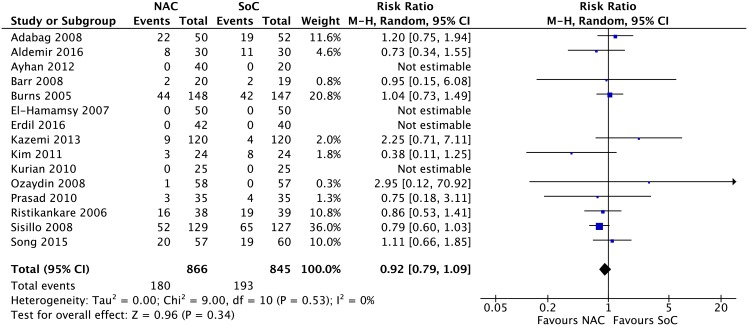
Meta-analysis on acute renal insufficiency.

**Fig 4:**
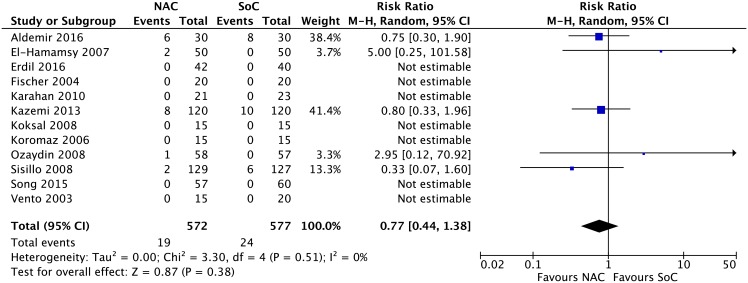
Meta-analysis on cardiac insufficiency.

**Fig 5:**
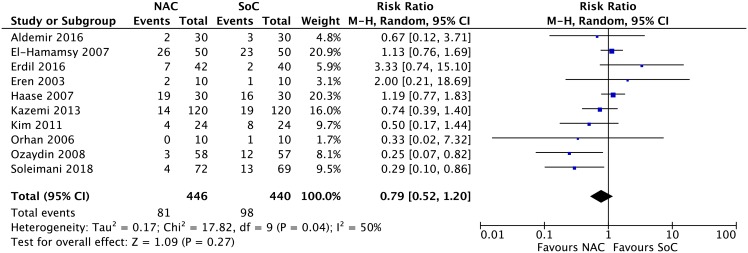
Meta-analysis on arrhythmia.

**Fig 6:**
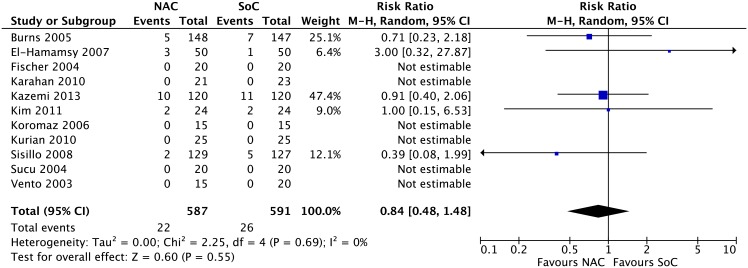
Meta-analysis on acute myocardial infarction.

**Clinical outcomes.** Results from 15 RCTs [52–56, 58, 59, 64, 65, 68, 70, 72, 73, 75, 77] with a total of 1,711 patients for acute renal insufficiency (RR 0.92, 95% CI 0.79 to 1.09; events (NAC:180/886, SoC:193/845); I^2^ = 0%; p = 0.34) (Fig 3); 12 RCTs [53, 58, 59, 61, 63, 64, 66, 67, 70, 75, 77, 78] with a total of 1,149 patients for cardiac insufficiency (RR 0.77, 95% CI 0.44 to 1.38; events (NAC:19/572; SoC:24/577); I^2^ = 0%; p = 0.38) (Fig 4); 10 RCTs [53, 58–60, 62, 64, 65, 69, 70, 76] with a total of 886 patients for arrhythmia (RR 0.79, 95% CI 0.52 to 1.20; events (NAC:81/446, SoC:98/440); I^2^ = 50%; p = 0.27) (Fig 5); and 11 RCTs [20, 56, 60, 61, 63, 64, 65, 67, 68, 75, 78] with a total of 1,178 patients for acute myocardial infarction (RR 0.84, 95% CI 0.48 to 1.48; events (NAC:22/587, SoC:26/591); I^2^ = 0%; p = 0.55) (Fig 6) yielded a non statistically significant difference between NAC and SoC in patients submitted to cardiac surgery.

The certainty of evidence was low for all outcomes due to very serious imprecision (low number of events and wide confidence intervals including clinically important benefit and harm) (Table 1). Based on funnel plot analyses, no publication bias was detected (S2 Fig) for any outcomes.

With respect to subgroups of interest, we found no statistically significant differences based on route, dose, timing of administration of NAC, surgical or anesthetic technique and characteristics of the population (S3 Fig, panel B,C, F, and G).

**Process outcomes.** Results from 18 RCTs [52, 53, 55, 56, 58, 59, 62, 63–65, 67, 69–72, 76–78] with a total of 1,650 patients for hospital length of stay (HLoS) (MD -0.21 days, 95% CI -0.64 to 0.23; I^2^ = 89%; p = 0.35) (S4 Fig, panel A); and 17 RCTs [52, 53, 55, 56, 59, 62–65, 67, 69, 71–73, 76–78] with a total of 1,512 patients for intensive care unit length of stay (ICULoS) (MD -0.04 days, 95% CI -0.29 to 0.20; I^2^ = 95%; p = 0.73) (S4 Fig, panel A), yielded a non statistically significant difference between NAC and SoC in patients submitted to cardiac surgery.

The certainty of evidence was rated as moderate for both outcomes due to inconsistency (Table 1) and no publication bias was detected (S2 Fig).

**Fig 7:**
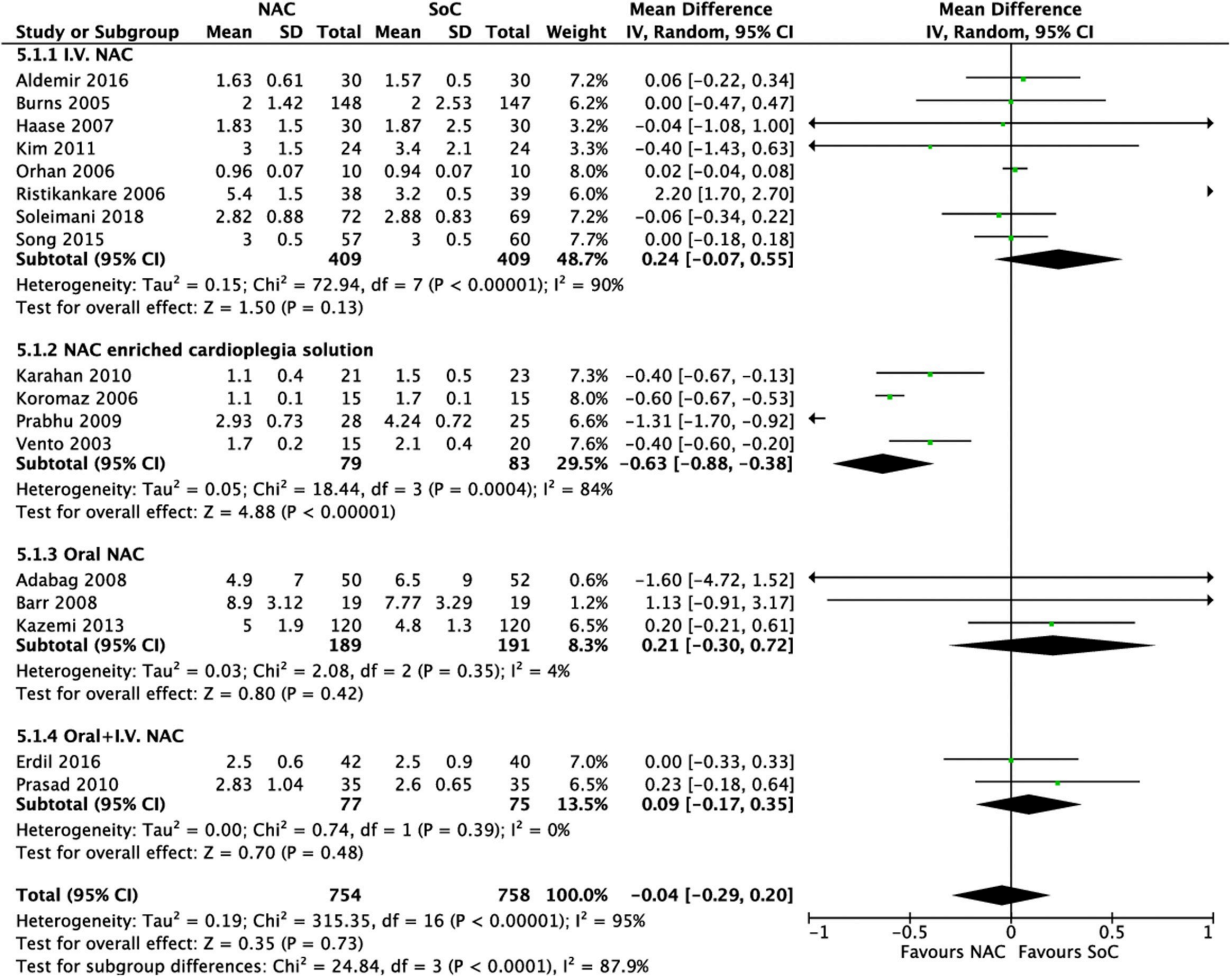
Meta-analysis on ICU and length of stay (days), according to the route of administration of NAC.

With respect to subgroups of interest, we found no statistically significant differences based on dose, timing of administration of NAC, or based on surgical or anesthetic technique and characteristics of the population (S3 Fig, panel D and E). However, regarding the a priori hypothesized subgroup based on route of administration, results from four RCTs [63, 67, 71, 78] suggested a statistically significant reduction with the use of enriched cardioplegia solution with NAC compared to SoC in intensive care unit length of stay (MD -0.63 days, 95% CI -0.88 to -0.38; n = 162; I^2^ = 84%; p < 0.00001) (Fig 7). We downgraded the certainty of evidence to low for imprecision as a result of a limited number of patients, and for inconsistency (overall I^2^ of 95% was reduced to 84% only, among those receiving NAC).

We also presented sensitivity analyses on low risk bias (S5 Fig). There was no statistically significant difference in any of our sensitivity analysis.

## Discussion

### Main findings

The main findings in this meta-analysis showed no statistically significant differences in mortality, acute renal insufficiency, acute cardiac insufficiency, HLoS, ICULoS, arrhythmia and myocardial infarction between NAC and SoC in cardiac surgery. However, the wide confidence intervals show that we cannot rule out either clinically significant benefit or harm with the use of NAC. Additionally, a sensitivity analysis including only the low risk of bias studies was performed for each of the outcomes and no significant differences emerged from this analysis.

Possible explanations for no statistically significant results in mortality rates include: a) glutathione resources might not have been depleted by the level of oxidative stress resulting from cardiac surgery; b) glutathione peroxidase enzyme might have lost its activity due to oxidative stress, making it irrelevant to replenish glutathione reserves; and c) it is possible that anesthetic drugs, such as propofol and halogenated anesthetics, may also have an antioxidant effect, blurring the difference between NAC and SoC [80].

Other factors that might have influenced the results include the wide variability of NAC treatment regimens in dose (4–300 mg/kg/day), duration (1 hour–5 days), and routes of administration (oral, intravenous, cardioplegia, oral plus intravenous.), causing the plasma concentration of NAC to vary widely as well, thus potentially influencing the results.

Although there were few losses to follow-up, the wide range of follow-up period (1–90 days) might have caused relevant data to be lost across the outcomes, especially for the long-term mortality rates.

Considering the reduction in ICULoS with the addition of NAC to the cardioplegia solution, it may be useful to assess the impact of NAC using this route of administration on the incidence of major outcomes in larger trials.

The overall certainty of evidence was rated low for mortality, ARI, ACI, arrhythmia and AMI primarily due to very serious issues of imprecision, and rated down to moderate for HLoS and ICULoS due to sample heterogeneity.

### Strengths and limitations

Our review has numerous strengths including an extensive and sensitive search of the literature on the subject, with no restrictions on language or publication status. We included revascularization, valve and combined surgeries with and without CPB and to our knowledge this is the most extensive search of the potential use of NAC as an adjunct to perioperative cardiac surgeries in the literature to date. We also included studies in which the administration of NAC was offered using different routes, dosages and durations, and we extracted and analyzed data on the seven main outcomes deemed most important for the cardiac surgery population. Furthermore, we independently rated the overall certainty of evidence using the GRADE approach for each outcome, and we pre-specified the subgroup analyses and assessed the results based on published assessment criteria [50], including the use of a test of interaction. We also assessed publication bias for the outcomes with ten or more studies included, with none of our funnel plots suggesting the possibility of publication bias.

The primary limitation of our review is the low certainty evidence based on the overall body of evidence. Despite the moderate number of identified trials (29), the amount of evidence is insufficient.

Another limitation of this review was related to the quality of the included studies. Overall, we rated the studies at a high risk of bias due to unblinding of participants [72] and personnel in eight trials [63, 65–68, 72, 20, 78]; and no reporting of allocation concealment in eight additional studies [20, 63, 65–68, 72, 78]. We conducted sensitivity analyses for each of our seven a priori outcomes to assess if there was a significant difference between studies at high versus low risk of bias, with results indicating no differences between subgroups (S7 Table).

Furthermore, the included RCTs were heterogeneous in terms of the population characteristics (e.g., trials that included only patients with high or low cardiac ejection fractions, or patients with or without kidney dysfunction) and intervention characteristics (e.g., trials differed with respect to routes, doses, duration of treatment, as well as different surgical and anesthetic techniques). In an attempt to overcome this limitation, we performed a priori subgroup analyses to explore different populations and intervention characteristics for each of our seven outcomes. After assessing for credibility using five published criteria [50], only one subgroup demonstrated a statistically significant test of interaction indicating that adding NAC to the cardioplegia solution reduces ICULoS in patients undergoing cardiac surgeries.

### Relation to previous studies

Six systematic reviews [26–31] have been published in the past 10 years relevant to our study objectives, with the most extensive systematic review having included 13 RCTs and 1,338 patients [26]. Compared to our review, all of the previous reviews included fewer outcomes and fewer RCTs. Five of these reviews excluded RCTs that did not match the primary outcomes to the reviews’ primary outcome sought [26, 28–31]. For instance, some reviews only included RCTs that had ARI as their primary outcome [26, 29, 30], while others only included studies in which the primary outcome was a single type of arrhythmia (atrial fibrillation) [28, 31].

Only one review [27] sought to analyze multiple critically important outcomes such as mortality, ARI, hospital length of stay and atrial fibrillation. However, unlike our review, RCTs where NAC was administered through the cardioplegia solution were excluded from this review.

Although all systematic reviews yielded results suggesting better outcomes with the use of NAC, only Ali-Hassan-Sayegh et al. (2014) [31] and Gu et al. (2012) [28] found statistically significant reductions in the incidence of arrhythmias. These authors however limited their results to atrial fibrillation, narrowing the scope to a single type of arrhythmia, among several possible postoperative arrhythmias (atrial fibrillation, atrial flutter, premature atrial complexes, multifocal atrial tachycardia, premature ventricular complexes, nonsustained ventricular tachycardia, ventricular tachycardia, sick sinus syndrome, and atrioventricular block) [32]. Our review assessed the protective antioxidant effect of NAC on inflammation and ischemia injuries through its impact on the overall incidence of arrhythmias.

### Clinical implications of the study

CPB and ischemia-reperfusion injury are associated with oxidative stress [49,81] and antioxidants play a protective role in cardiac surgery [19]. NAC has antioxidant effects by regenerating glutathione [82] and its protective effects are more evident after parenteral administration [46].

Although our results were generally rated as low (some as moderate) certainty evidence and did not find a statistically significant difference on major outcomes when NAC was added to the treatment of patients undergoing cardiac surgery, it was safely administered to patients in all included studies in this review.

### Research implications of the study

Based on the data from this systematic review, and after conducting a sample size calculation to determine the number of participants needed to definitively determine the potential efficacy of NAC for all-cause mortality, based on a 2.3% risk of mortality from the NAC group and a 3.9% risk of mortality derived from over 20,000 patients studied by Nashef’s [83] using an alpha value 5% and beta of 20%, a future study trial would need to randomize at least 3,682 patients [84].

## Conclusions

This comprehensive meta-analysis of 29 RCTs provides current evidence for whether or not to add NAC to the treatment of patients in the perioperative period during cardiac surgery. It confirms previous observations that NAC can be safely administered to patients, but fails to demonstrate significant efficacy in reducing major adverse outcomes associated with cardiac surgeries.

## Supporting information

S1 Fig Risk of bias assessment. (TIFF)

S2 Fig Funnel plots of clinical outcomes. (DOCX)

S3 Fig Sensitivity analyses on low risk bias. Panel A. Mortality. Panel B. Acute renal insufficiency. Panel C. Cardiac insufficiency. Panel D. Hospital length of stay. Panel E. ICU length of stay. Panel F. Arrhythmia. Panel G. Acute myocardial infarction.
(ZIP)

S4 Fig Meta-analyses of process outcomes. (PDF)

S5 Fig Sensitivity analyses on low risk bias. (PDF)

S1 Table Search strategy in PubMed. (DOCX)

S2 Table Search strategy in Anesthesia and Analgesia. (DOCX)

S3 Table Data extraction form. (DOC)

S4 Table Excluded studies with reasons. (DOCX)

S5 Table Study characteristics related to population and setting. (DOCX)

S6 Table Study characteristics related to intervention and control groups. (DOCX)

S7 Table Risk of bias assessment. (DOCX)

S8 Table PRISMA checklist. (DOC)
