# Supplementary figures and images for: N-acetylcysteine use among patients undergoing cardiac surgery: A systematic review and meta-analysis of randomized trials
Source: PLoS One. 2019 May 9;14(5):e0213862. doi: 10.1371/journal.pone.0213862 (PMC6508704; doi:10.1371/journal.pone.0213862)

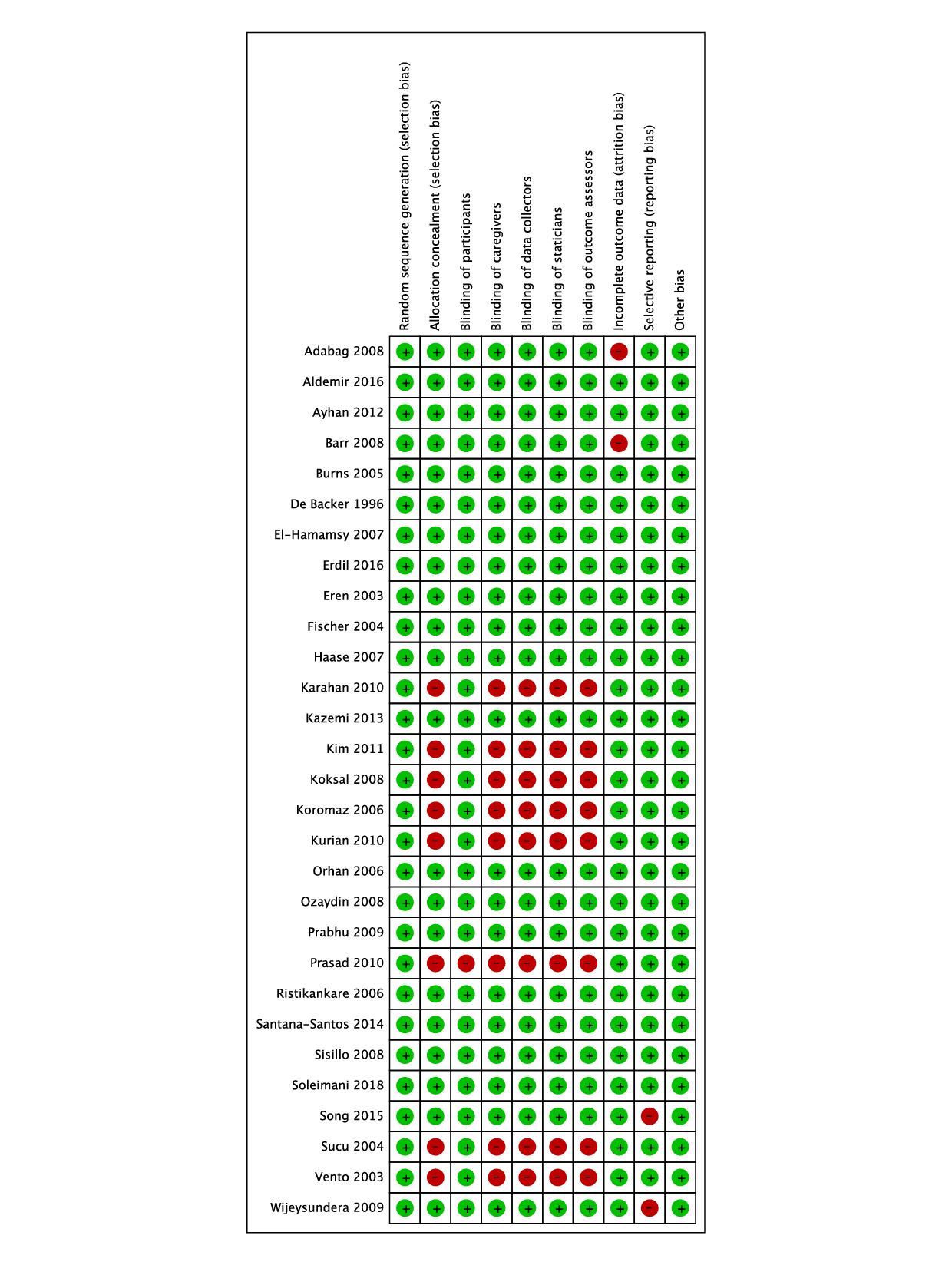

Supplement: S1 Fig — (TIFF) [file pone.0213862.s001.tiff]

Panel A

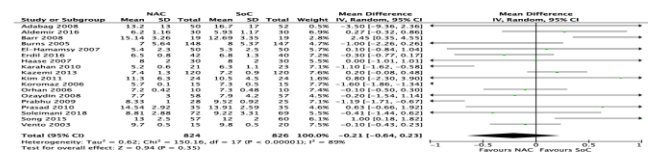

Panel B

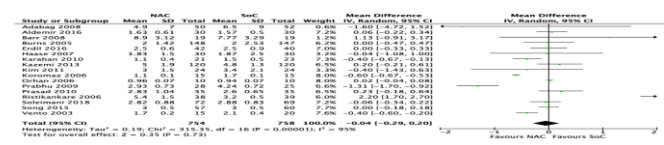

Supplement: S4 Fig — (PDF) [file pone.0213862.s004.pdf]
