# Supplementary material for: N-acetylcysteine use among patients undergoing cardiac surgery: A systematic review and meta-analysis of randomized trials
Source: PLoS One. 2019 May 9;14(5):e0213862. doi: 10.1371/journal.pone.0213862 (PMC6508704; doi:10.1371/journal.pone.0213862)
Supplement: S2 Fig — (DOCX) [file pone.0213862.s002.docx]

**Figure S2**. Funnel plots of clinical outcomes.

| Panel A. Mortality  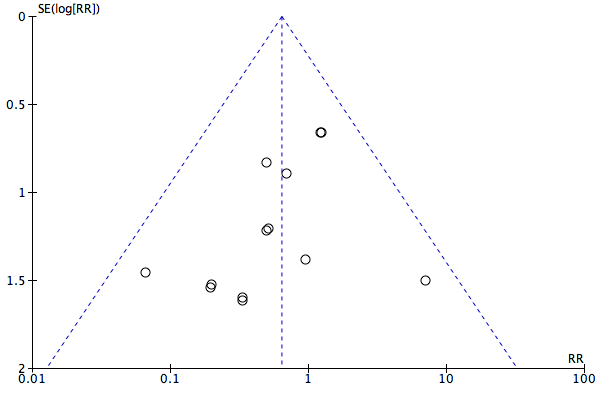 | Panel B. Acute renal insufficiency  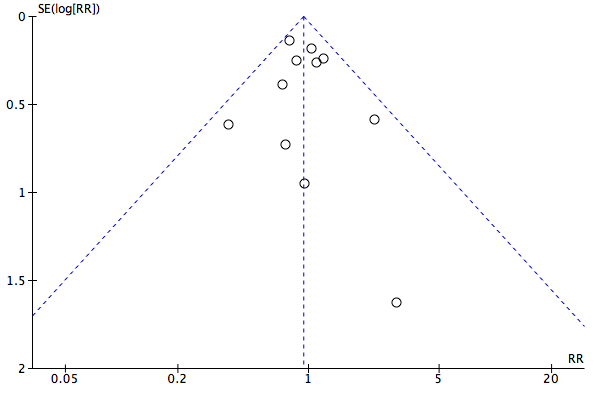 |
| --- | --- |
| Panel C. Hospital length of stay  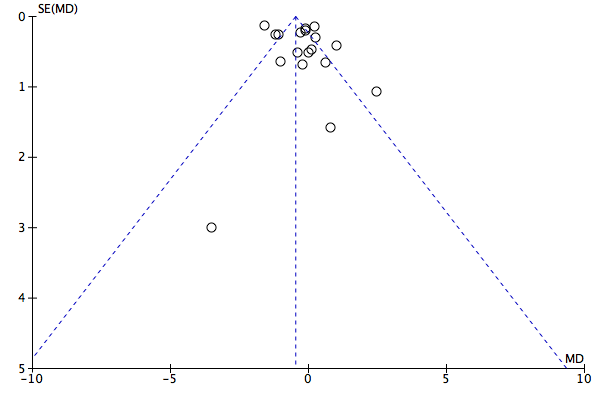 | Panel D. Intensive care unit length of stay  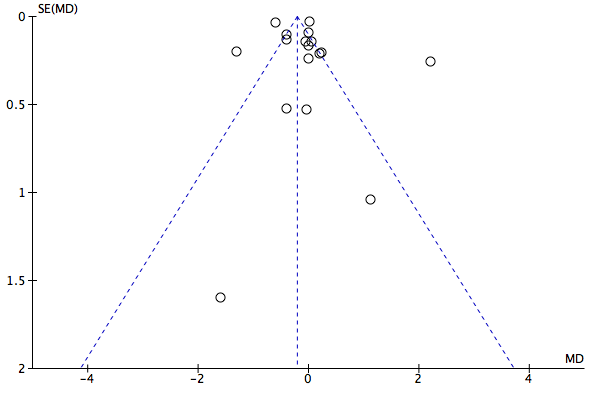 |
| Panel E. Arrhythmia  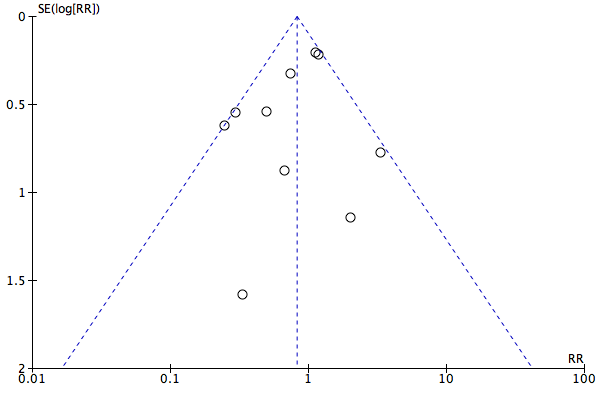 |  |
|  |  |
