# Supplementary material for: N-acetylcysteine use among patients undergoing cardiac surgery: A systematic review and meta-analysis of randomized trials
Source: PLoS One. 2019 May 9;14(5):e0213862. doi: 10.1371/journal.pone.0213862 (PMC6508704; doi:10.1371/journal.pone.0213862)
Supplement: S3 Fig — Panel A. Mortality. Panel B. Acute renal insufficiency. Panel C. Cardiac insufficiency. Panel D. Hospital length of stay. Panel E. ICU length of stay. Panel F. Arrhythmia. Panel G. Acute myocardial infarction. (ZIP) [file pone.0213862.s003.zip › S3 Fig. Subgroup analysis /S3 Fig. Subgroup analysis. Panel A. Mortality.docx]

**S3 Fig, Panel A. Mortality**. Subgroup analysis on clinical outcomes according to route, dose and duration of NAC, respectively.

| \| 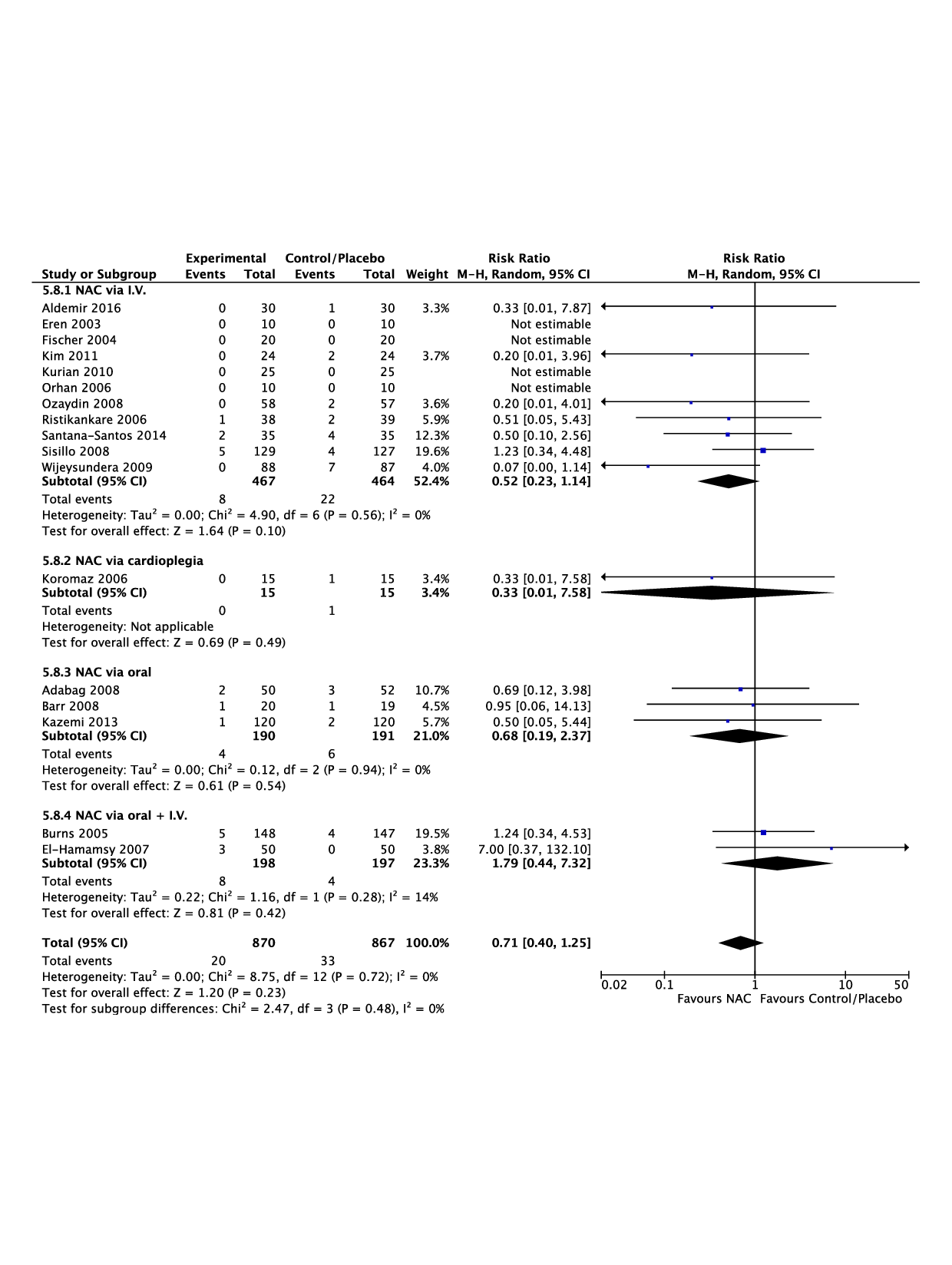 \| 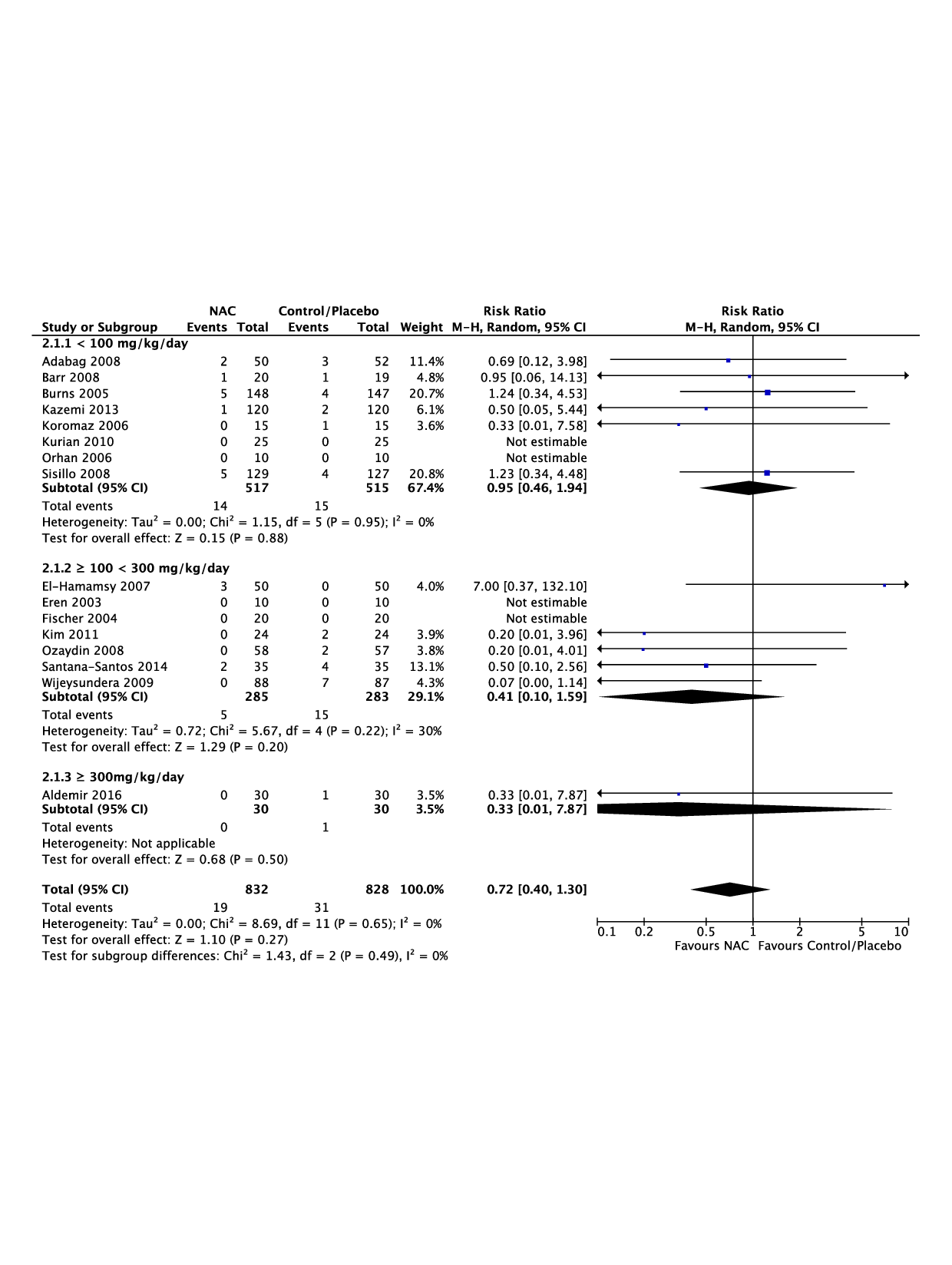 \| 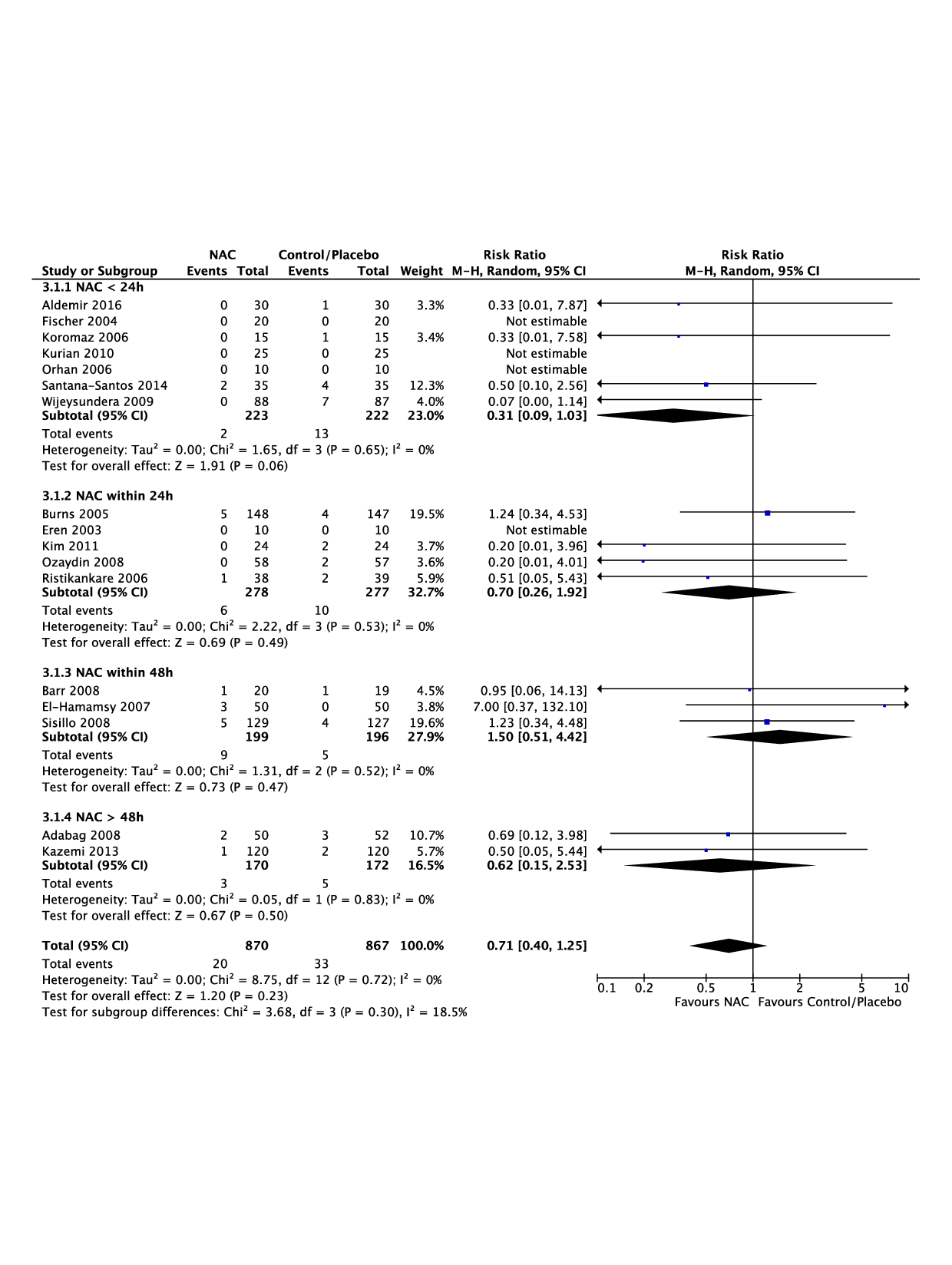 \| \| --- \| --- \| --- \| |  |
| --- | --- | --- | --- | --- |
