# Supplementary material for: N-acetylcysteine use among patients undergoing cardiac surgery: A systematic review and meta-analysis of randomized trials
Source: PLoS One. 2019 May 9;14(5):e0213862. doi: 10.1371/journal.pone.0213862 (PMC6508704; doi:10.1371/journal.pone.0213862)

*Panel A. Mortality*

| Study or Subgroup | NAC Events | NAC Total | SoC Events | SoC Total | Weight | Risk Ratio M-H, Random, 95% CI |
|---|---|---|---|---|---|---|
| Adabag 2008 | 2 | 50 | 3 | 52 | 11.5% | 0.69 [0.12, 3.98] |
| Aldemir 2016 | 0 | 30 | 1 | 30 | 3.5% | 0.33 [0.01, 7.87] |
| Barr 2008 | 1 | 20 | 1 | 19 | 4.8% | 0.95 [0.06, 14.13] |
| Burns 2005 | 5 | 148 | 4 | 147 | 21.0% | 1.24 [0.34, 4.53] |
| El-Hamamsy 2007 | 3 | 50 | 0 | 50 | 4.1% | 7.00 [0.37, 132.10] |
| Eren 2003 | 0 | 10 | 0 | 10 | | Not estimable |
| Fischer 2004 | 0 | 20 | 0 | 20 | | Not estimable |
| Kazemi 2013 | 1 | 120 | 2 | 120 | 6.2% | 0.50 [0.05, 5.44] |
| Kim 2011 | 0 | 24 | 2 | 24 | 0.0% | 0.20 [0.01, 3.96] |
| Koromaz 2006 | 0 | 15 | 1 | 15 | 0.0% | 0.33 [0.01, 7.58] |
| Kurian 2010 | 0 | 25 | 0 | 25 | | Not estimable |
| Orhan 2006 | 0 | 10 | 0 | 10 | | Not estimable |
| Ozaydin 2008 | 0 | 58 | 2 | 57 | 3.9% | 0.20 [0.01, 4.01] |
| Ristikankare 2006 | 1 | 38 | 2 | 39 | 6.3% | 0.51 [0.05, 5.43] |
| Santana-Santos 2014 | 2 | 35 | 4 | 35 | 13.2% | 0.50 [0.10, 2.56] |
| Sisillo 2008 | 5 | 129 | 4 | 127 | 21.1% | 1.23 [0.34, 4.48] |
| Wijeysundera 2009 | 0 | 88 | 7 | 87 | 4.3% | 0.07 [0.00, 1.14] |
| **Total (95% CI)** | | **806** | | **803** | **100.0%** | **0.76 [0.42, 1.38]** |
| Total events | 20 | | 30 | | | |

Heterogeneity: $Tau^2 = 0.00$; $Chi^2 = 7.78$, df = 10 (P = 0.65); $I^2 = 0\%$
Test for overall effect: Z = 0.90 (P = 0.37)

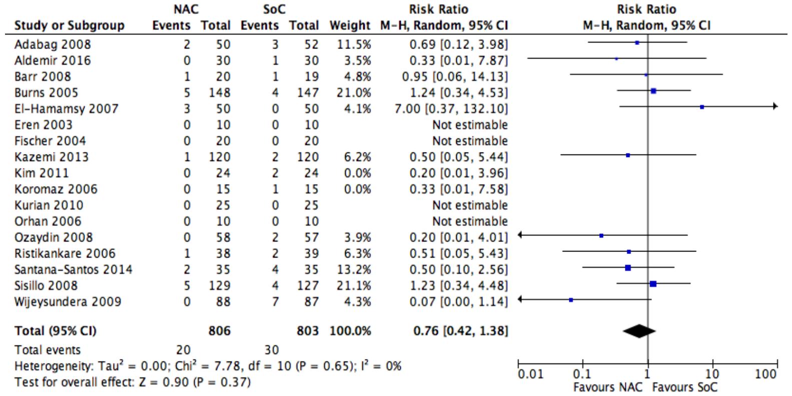


*Panel B. Acute renal insufficiency*

| Study or Subgroup | NAC Events | NAC Total | SoC Events | SoC Total | Weight | Risk Ratio M-H, Random, 95% CI |
|---|---|---|---|---|---|---|
| Adabag 2008 | 22 | 50 | 19 | 52 | 12.0% | 1.20 [0.75, 1.94] |
| Aldemir 2016 | 8 | 30 | 11 | 30 | 4.7% | 0.73 [0.34, 1.55] |
| Ayhan 2012 | 0 | 40 | 0 | 20 | | Not estimable |
| Barr 2008 | 2 | 20 | 2 | 19 | 0.8% | 0.95 [0.15, 6.08] |
| Burns 2005 | 44 | 148 | 42 | 147 | 21.4% | 1.04 [0.73, 1.49] |
| El-Hamamsy 2007 | 0 | 50 | 0 | 50 | | Not estimable |
| Erdil 2016 | 0 | 42 | 0 | 40 | | Not estimable |
| Kazemi 2013 | 9 | 120 | 4 | 120 | 2.1% | 2.25 [0.71, 7.11] |
| Kim 2011 | 3 | 24 | 8 | 24 | 0.0% | 0.38 [0.11, 1.25] |
| Kurian 2010 | 0 | 25 | 0 | 25 | | Not estimable |
| Ozaydin 2008 | 1 | 58 | 0 | 57 | 0.3% | 2.95 [0.12, 70.92] |
| Prasad 2010 | 3 | 35 | 4 | 35 | 0.0% | 0.75 [0.18, 3.11] |
| Ristikankare 2006 | 16 | 38 | 19 | 39 | 11.2% | 0.86 [0.53, 1.41] |
| Sisillo 2008 | 52 | 129 | 65 | 127 | 37.2% | 0.79 [0.60, 1.03] |
| Song 2015 | 20 | 57 | 19 | 60 | 10.3% | 1.11 [0.66, 1.85] |
| **Total (95% CI)** | | **782** | | **761** | **100.0%** | **0.94 [0.80, 1.11]** |
| Total events | 174 | | 181 | | | |

Heterogeneity: $Tau^2 = 0.00$; $Chi^2 = 6.75$, df = 8 (P = 0.56); $I^2 = 0\%$
Test for overall effect: Z = 0.71 (P = 0.48)

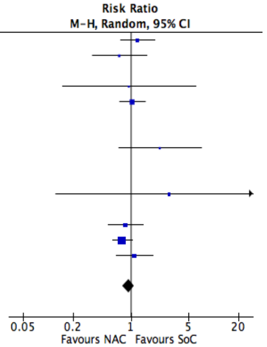


*Panel C. Cardiac insufficiency*

| Study or Subgroup | NAC Events | NAC Total | SoC Events | SoC Total | Weight | Risk Ratio M-H, Random, 95% CI |
|---|---|---|---|---|---|---|
| Aldemir 2016 | 6 | 30 | 8 | 30 | 38.4% | 0.75 [0.30, 1.90] |
| El-Hamamsy 2007 | 2 | 50 | 0 | 50 | 3.7% | 5.00 [0.25, 101.58] |
| Erdil 2016 | 0 | 42 | 0 | 40 | | Not estimable |
| Fischer 2004 | 0 | 20 | 0 | 20 | | Not estimable |
| Karahan 2010 | 0 | 21 | 0 | 23 | | Not estimable |
| Kazemi 2013 | 8 | 120 | 10 | 120 | 41.4% | 0.80 [0.33, 1.96] |
| Koksal 2008 | 0 | 15 | 0 | 15 | | Not estimable |
| Koromaz 2006 | 0 | 15 | 0 | 15 | | Not estimable |
| Ozaydin 2008 | 1 | 58 | 0 | 57 | 3.3% | 2.95 [0.12, 70.92] |
| Sisillo 2008 | 2 | 129 | 6 | 127 | 13.3% | 0.33 [0.07, 1.60] |
| Song 2015 | 0 | 57 | 0 | 60 | | Not estimable |
| Vento 2003 | 0 | 15 | 0 | 20 | | Not estimable |
| **Total (95% CI)** | | **572** | | **577** | **100.0%** | **0.77 [0.44, 1.38]** |
| Total events | 19 | | 24 | | | |

Heterogeneity: $Tau^2 = 0.00$; $Chi^2 = 3.30$, df = 4 (P = 0.51); $I^2 = 0\%$
Test for overall effect: Z = 0.87 (P = 0.38)

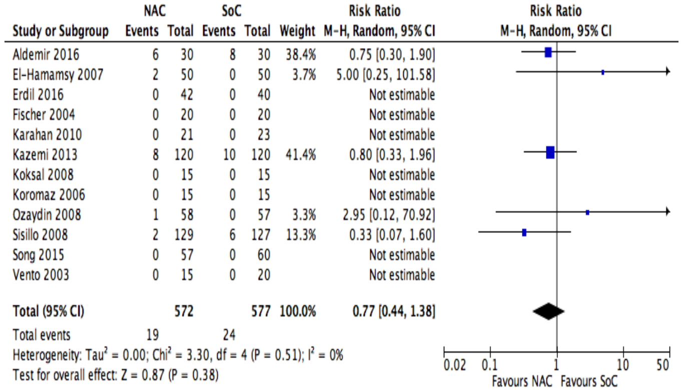


*Panel D. Hospital length of stay*

| Study or Subgroup | NAC Mean | NAC SD | NAC Total | SoC Mean | SoC SD | SoC Total | Weight | Mean Difference IV, Random, 95% CI |
|---|---|---|---|---|---|---|---|---|
| Adabag 2008 | 13.2 | 13 | 50 | 16.7 | 17 | 52 | 0.4% | -3.50 [-9.36, 2.36] |
| Aldemir 2016 | 6.2 | 1.16 | 30 | 5.93 | 1.17 | 30 | 10.3% | 0.27 [-0.32, 0.86] |
| Barr 2008 | 15.14 | 3.26 | 19 | 12.69 | 3.35 | 19 | 2.5% | 2.45 [0.35, 4.55] |
| Burns 2005 | 7 | 5.64 | 148 | 8 | 5.37 | 147 | 5.3% | -1.00 [-2.26, 0.26] |
| El-Hamamsy 2007 | 5.4 | 2.3 | 50 | 5.3 | 2.5 | 50 | 7.3% | 0.10 [-0.84, 1.04] |
| Erdil 2016 | 6.5 | 0.8 | 42 | 6.8 | 1.3 | 40 | 11.4% | -0.30 [-0.77, 0.17] |
| Haase 2007 | 8 | 2 | 30 | 8 | 2 | 30 | 6.8% | 0.00 [-1.01, 1.01] |
| Karahan 2010 | 5.2 | 0.6 | 21 | 6.3 | 1.1 | 23 | 0.0% | -1.10 [-1.62, -0.58] |
| Kazemi 2013 | 7.4 | 1.3 | 120 | 7.2 | 0.9 | 120 | 13.0% | 0.20 [-0.08, 0.48] |
| Kim 2011 | 11.3 | 6.3 | 24 | 10.5 | 4.5 | 24 | 0.0% | 0.80 [-2.30, 3.90] |
| Koromaz 2006 | 5.7 | 0.1 | 15 | 7.3 | 0.5 | 15 | 0.0% | -1.60 [-1.86, -1.34] |
| Orhan 2006 | 7.2 | 0.42 | 10 | 7.3 | 0.48 | 10 | 12.1% | -0.10 [-0.50, 0.30] |
| Ozaydin 2008 | 7.7 | 3 | 58 | 7.9 | 4.2 | 57 | 4.9% | -0.20 [-1.54, 1.14] |
| Prabhu 2009 | 8.33 | 1 | 28 | 9.52 | 0.92 | 25 | 11.0% | -1.19 [-1.71, -0.67] |
| Prasad 2010 | 14.54 | 2.92 | 35 | 13.91 | 2.59 | 35 | 0.0% | 0.63 [-0.66, 1.92] |
| Soleimani 2018 | 8.81 | 2.88 | 72 | 9.22 | 3.31 | 69 | 6.7% | -0.41 [-1.44, 0.62] |
| Song 2015 | 13 | 2.5 | 57 | 12 | 2 | 60 | 8.2% | 1.00 [0.18, 1.82] |
| Vento 2003 | 9.7 | 0.5 | 15 | 9.8 | 0.5 | 20 | 0.0% | -0.10 [-0.43, 0.23] |
| **Total (95% CI)** | | | **714** | | | **709** | **100.0%** | **-0.08 [-0.44, 0.29]** |

Heterogeneity: $Tau^2 = 0.25$; $Chi^2 = 39.78$, df = 12 (P < 0.0001); $I^2 = 70\%$
Test for overall effect: Z = 0.41 (P = 0.68)

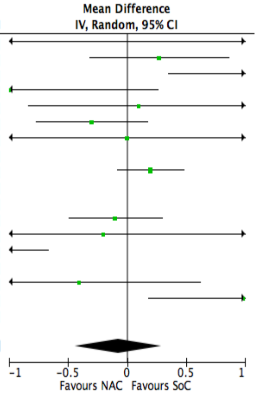

## *Panel E. Intensive care unit length of stay*

| | NAC | | | SoC | | | | Mean Difference |
|---|---|---|---|---|---|---|---|---|
| **Study or Subgroup** | **Mean** | **SD** | **Total** | **Mean** | **SD** | **Total** | **Weight** | **IV, Random, 95% CI** |
| Adabag 2008 | 4.9 | 7 | 50 | 6.5 | 9 | 52 | 0.8% | −1.60 [−4.72, 1.52] |
| Aldemir 2016 | 1.63 | 0.61 | 30 | 1.57 | 0.5 | 30 | 10.7% | 0.06 [−0.22, 0.34] |
| Barr 2008 | 8.9 | 3.12 | 19 | 7.77 | 3.29 | 19 | 1.8% | 1.13 [−0.91, 3.17] |
| Burns 2005 | 2 | 1.42 | 148 | 2 | 2.53 | 147 | 9.2% | 0.00 [−0.47, 0.47] |
| Erdil 2016 | 2.5 | 0.6 | 42 | 2.5 | 0.9 | 40 | 10.3% | 0.00 [−0.33, 0.33] |
| Haase 2007 | 1.83 | 1.5 | 30 | 1.87 | 2.5 | 30 | 4.8% | −0.04 [−1.08, 1.00] |
| Karahan 2010 | 1.1 | 0.4 | 21 | 1.5 | 0.5 | 23 | 0.0% | −0.40 [−0.67, −0.13] |
| Kazemi 2013 | 5 | 1.9 | 120 | 4.8 | 1.3 | 120 | 9.7% | 0.20 [−0.21, 0.61] |
| Kim 2011 | 3 | 1.5 | 24 | 3.4 | 2.1 | 24 | 0.0% | −0.40 [−1.43, 0.63] |
| Koromaz 2006 | 1.1 | 0.1 | 15 | 1.7 | 0.1 | 15 | 0.0% | −0.60 [−0.67, −0.53] |
| Orhan 2006 | 0.96 | 0.07 | 10 | 0.94 | 0.07 | 10 | 11.8% | 0.02 [−0.04, 0.08] |
| Prabhu 2009 | 2.93 | 0.73 | 28 | 4.24 | 0.72 | 25 | 9.9% | −1.31 [−1.70, −0.92] |
| Prasad 2010 | 2.83 | 1.04 | 35 | 2.6 | 0.65 | 35 | 0.0% | 0.23 [−0.18, 0.64] |
| Ristikankare 2006 | 5.4 | 1.5 | 38 | 3.2 | 0.5 | 39 | 8.9% | 2.20 [1.70, 2.70] |
| Soleimani 2018 | 2.82 | 0.88 | 72 | 2.88 | 0.83 | 69 | 10.7% | −0.06 [−0.34, 0.22] |
| Song 2015 | 3 | 0.5 | 57 | 3 | 0.5 | 60 | 11.4% | 0.00 [−0.18, 0.18] |
| Vento 2003 | 1.7 | 0.2 | 15 | 2.1 | 0.4 | 20 | 0.0% | −0.40 [−0.60, −0.20] |
| **Total (95% CI)** | | | **644** | | | **641** | **100.0%** | **0.09 [−0.20, 0.39]** |

Heterogeneity: $Tau^2 = 0.19$; $Chi^2 = 120.27$, df = 11 (P < 0.00001); $I^2 = 91\%$
Test for overall effect: Z = 0.61 (P = 0.54)

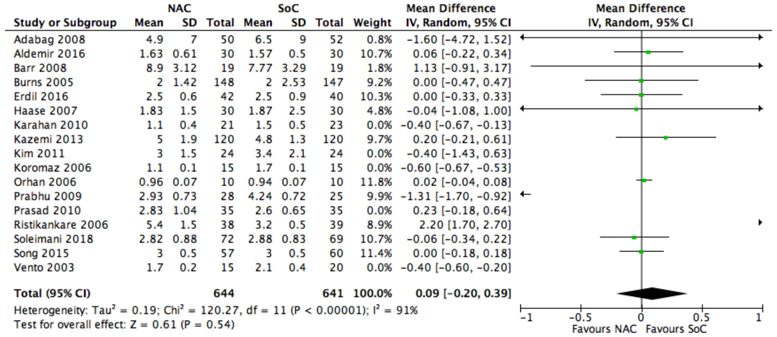


## *Panel F. Arrhythmia*

| | NAC | | SoC | | | Risk Ratio |
|---|---|---|---|---|---|---|
| **Study or Subgroup** | **Events** | **Total** | **Events** | **Total** | **Weight** | **M-H, Random, 95% CI** |
| Aldemir 2016 | 2 | 30 | 3 | 30 | 5.4% | 0.67 [0.12, 3.71] |
| El-Hamamsy 2007 | 26 | 50 | 23 | 50 | 23.0% | 1.13 [0.76, 1.69] |
| Erdil 2016 | 7 | 42 | 2 | 40 | 6.6% | 3.33 [0.74, 15.10] |
| Eren 2003 | 2 | 10 | 1 | 10 | 3.4% | 2.00 [0.21, 18.69] |
| Haase 2007 | 19 | 30 | 16 | 30 | 22.3% | 1.19 [0.77, 1.83] |
| Kazemi 2013 | 14 | 120 | 19 | 120 | 17.7% | 0.74 [0.39, 1.40] |
| Kim 2011 | 4 | 24 | 8 | 24 | 0.0% | 0.50 [0.17, 1.44] |
| Orhan 2006 | 0 | 10 | 1 | 10 | 1.9% | 0.33 [0.02, 7.32] |
| Ozaydin 2008 | 3 | 58 | 12 | 57 | 9.1% | 0.25 [0.07, 0.82] |
| Soleimani 2018 | 4 | 72 | 13 | 69 | 10.6% | 0.29 [0.10, 0.86] |
| **Total (95% CI)** | | **422** | | **416** | **100.0%** | **0.83 [0.53, 1.29]** |
| Total events | 77 | | 90 | | | |

Heterogeneity: $Tau^2 = 0.18$; $Chi^2 = 16.37$, df = 8 (P = 0.04); $I^2 = 51\%$
Test for overall effect: Z = 0.82 (P = 0.41)

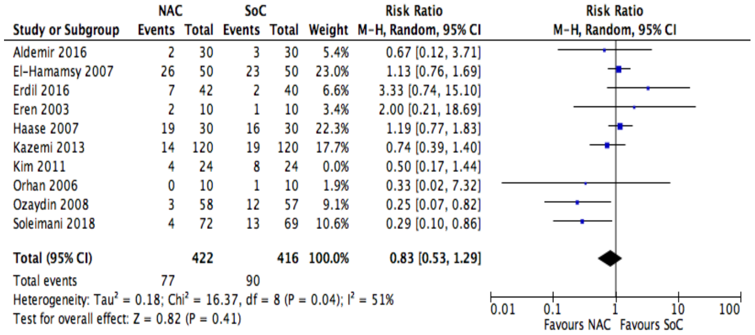


## *Panel G. Acute myocardial infarction*

| | NAC | | SoC | | | Risk Ratio |
|---|---|---|---|---|---|---|
| **Study or Subgroup** | **Events** | **Total** | **Events** | **Total** | **Weight** | **M-H, Random, 95% CI** |
| Burns 2005 | 5 | 148 | 7 | 147 | 27.6% | 0.71 [0.23, 2.18] |
| El-Hamamsy 2007 | 3 | 50 | 1 | 50 | 7.0% | 3.00 [0.32, 27.87] |
| Fischer 2004 | 0 | 20 | 0 | 20 | | Not estimable |
| Karahan 2010 | 0 | 21 | 0 | 23 | | Not estimable |
| Kazemi 2013 | 10 | 120 | 11 | 120 | 52.1% | 0.91 [0.40, 2.06] |
| Kim 2011 | 2 | 24 | 2 | 24 | 0.0% | 1.00 [0.15, 6.53] |
| Koromaz 2006 | 0 | 15 | 0 | 15 | | Not estimable |
| Kurian 2010 | 0 | 25 | 0 | 25 | | Not estimable |
| Sisillo 2008 | 2 | 129 | 5 | 127 | 13.3% | 0.39 [0.08, 1.99] |
| Sucu 2004 | 0 | 20 | 0 | 20 | | Not estimable |
| Vento 2003 | 0 | 15 | 0 | 20 | | Not estimable |
| **Total (95% CI)** | | **467** | | **464** | **100.0%** | **0.83 [0.46, 1.49]** |
| Total events | 20 | | 24 | | | |

Heterogeneity: $Tau^2 = 0.00$; $Chi^2 = 2.21$, df = 3 (P = 0.53); $I^2 = 0\%$
Test for overall effect: Z = 0.63 (P = 0.53)

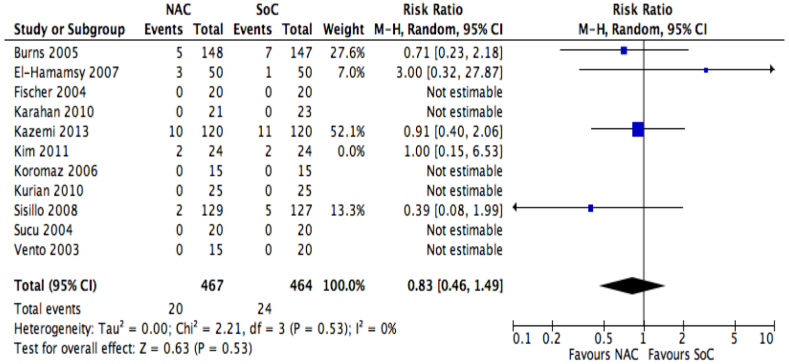

Supplement: S5 Fig — (PDF) [file pone.0213862.s005.pdf]
