# Supplementary material for: N-acetylcysteine use among patients undergoing cardiac surgery: A systematic review and meta-analysis of randomized trials
Source: PLoS One. 2019 May 9;14(5):e0213862. doi: 10.1371/journal.pone.0213862 (PMC6508704; doi:10.1371/journal.pone.0213862)
Supplement: S1 Table — (DOCX) [file pone.0213862.s006.docx]

**Table S1**. Search strategy in PubMed.

| (Coronary Artery Disease OR Coronary Artery Diseases OR Coronary Arteriosclerosis OR Coronary Arterioscleroses OR Coronary Atheroscleroses OR Coronary Atherosclerosis OR Cardiopulmonary Bypass OR Heart-Lung Bypass OR Heart Lung Bypass OR Heart-Lung Bypasses OR Cardiopulmonary Bypasses OR Bypass Surgery, Coronary Artery OR Bypass, Coronary Artery OR Internal Mammary Artery Implantation OR Myocardial Revascularizations OR Myocardial Revascularization OR CABG OR Coronary artery bypass graft OR Coronary artery bypass surgery OR Coronary Artery Bypasses OR Aortocoronary Bypass OR Aortocoronary Bypasses OR Coronary Artery Bypass Grafting OR Coronary Disease/surgery OR Coronary Vessels/surgery OR Myocardial Infarct/surgery OR Thoracic Surgery OR Thoracic Surgical Procedures OR Cardiovascular Surgical Procedures OR Cardiac Surgical Procedures OR Cardiac surgery OR Surgery, Thoracic OR Surgery, Heart OR Surgery, Cardiac OR Heart Surgery) **AND** (Acetylcysteine OR N-Acetyl-L-cysteine OR N Acetyl L cysteine OR N-Acetylcysteine OR N Acetylcysteine OR NAC OR N-AC OR N-acetyl cysteine OR Acemuc OR Acetabs OR Acetylcystein AL OR Acetylcystein Atid OR Acetylcystein Heumann OR Acetylcystein Trom OR Acetylcysteine Hydrochloride OR Acetylcysteine Sodium OR Acetylcysteine Zinc OR Acetylcysteine, (D)-Isomer OR Acetylcysteine, (DL)-Isomer OR Acetylcysteine, Monoammonium Salt OR Acetylcysteine, Monosodium Salt OR Acetylin OR Acetyst OR Acétylcystéine GNR OR Airbron OR Alveolex OR Azubronchin OR Bisolvon NAC OR Bromuc OR Broncho-Fips OR Broncholysin OR Broncoclar OR Codotussyl OR Cystamucil OR Dampo Mucopect OR Eurespiran OR Exomuc OR Fabrol OR Fluimucil OR Fluprowit OR Frekatuss OR Genac OR Hoestil OR Ilube OR Jenacystein OR Jenapharm OR Lantamed OR Larylin NAC OR Lindocetyl OR M-Pectil OR Mercapturic Acid OR Muciteran OR Muco Sanigen OR Mucomyst OR Mucosil OR Mucosol OR Mucosolvin OR NAC AL OR NAC Zambon OR Optipect Hustengetränk OR Siccoral OR Siran OR Solmucol OR acebraus OR durabronchal OR mentopin Acetylcystein) |
| --- |

^*^Search strategy restricted to RCTs and humans.
