# Supplementary material for: N-acetylcysteine use among patients undergoing cardiac surgery: A systematic review and meta-analysis of randomized trials
Source: PLoS One. 2019 May 9;14(5):e0213862. doi: 10.1371/journal.pone.0213862 (PMC6508704; doi:10.1371/journal.pone.0213862)
Supplement: S2 Table — (DOCX) [file pone.0213862.s007.docx]

**Table S2**. Search strategy in Anesthesia and Analgesia.

| acetylcysteiene AND cardiac surgery, acetylcysteine AND heart surgery, acetylcysteine AND coronary artery bypass graft surgery, acetylcysteine AND cardiac revascularization surgery, acetylcysteine AND heart valve surgery |
| --- |
