# Supplementary material for: N-acetylcysteine use among patients undergoing cardiac surgery: A systematic review and meta-analysis of randomized trials
Source: PLoS One. 2019 May 9;14(5):e0213862. doi: 10.1371/journal.pone.0213862 (PMC6508704; doi:10.1371/journal.pone.0213862)
Supplement: S3 Table — (DOC) [file pone.0213862.s008.doc]

# **Table S3.** Data extraction form.

| **Study ID (Author/year)** |
| --- |
|  |

## General Information

| **Report title**  *(title of paper/ abstract/ report that data are extracted from)* |  |
| --- | --- |
| **Report ID**  *(ID for this paper/ abstract/ report)* |  |
| **Publication type**  *(e.g. full report, abstract, letter)* |  |
| **Possible conflicts of interest**  *(for study authors)* |  |
| **Country** |  |

## Study Eligibility

| **Study Characteristics** | **Eligibility sriteria**  *(Insert eligibility criteria for each characteristic as defined in the Protocol)* |
| --- | --- |
| **Type of study** |  |
| **Participants** |  |
| **Inclusion criteria** |  |
| **Exclusion criteria** |  |
| **Types of intervention** |  |
| **Outcomes** |  |
| **Follow-up time** |  |

## Participants

|  | **Description as stated in report/paper** | |
| --- | --- | --- |
| **Total no. randomized**  *(or total pop. at start of study for NRCTs)* | NAC | Control |
| **Clusters**  *(if applicable, no., type, no. people per cluster)* |  | |
| **Withdrawals and exclusions**  *(if not provided below by outcome)* |  | |
| **Age (Mean/SD)** | NAC | Control |
| **Sex (Male)** | NAC | Control |
| **Co-morbidities** |  | |
| **Subgroups**  **(EF/renal dysfunction)** | EF (>0.4/<0.4) | Renal dysfunction (yes/no) |

## Intervention

|  | **Description as stated in report/paper** |
| --- | --- |
| **NAC regimen description**  **(dose/route/duration):** | Dose: |
|  | Route: |
|  | Duration: |
| **Surgical technique**  **(on-pump/off-pump)** |  |
| **Anesthesia technique**  **(TIVA/inhaled anesthetics)** |  |
| **Anticoagulation** |  |
| **Anticoagulation reversal** |  |
| **Measured outcomes** |  |
| **Co-morbidities** |  |
| **Other treatment received** *(additional to study intervention)* |  |
| **Other relevant sociodemographics** |  |
| **Subgroups measured** |  |
| **Subgroups reported** |  |

## Risk of Bias assessment

| **Domain** | **Risk of bias** | | | **Support for judgement** |
| --- | --- | --- | --- | --- |
|  | Definetely ow risk | Probably low / high | Definetely high risk |  |
| **Random sequence generation**  *(selection bias)* |  |  |  |  |
| **Allocation concealment**  *(selection bias)* |  |  |  |  |
| **Blinding of participants and personnel**  *(performance bias)* |  |  |  |  |
| **Blinding of caregivers** |  |  |  |  |
| **Blinding of data collectors** |  |  |  |  |
| **Blinding of staticians** |  |  |  |  |
| **Blinding of outcome assessment** |  |  |  |  |
| **Incomplete outcome data**  *(attrition bias)* |  |  |  | **Loss to follow-up:** |
| **Selective outcome reporting?**  *(reporting bias)* |  |  |  |  |
| **Other bias** |  |  |  |  |

## Results

Dichotomous outcome

|  | **Description as stated in report/paper** | | | |
| --- | --- | --- | --- | --- |
| **Comparison** |  | | | |
| **Outcome** |  | | | |
| **Subgroup** |  | | | |
| **Timepoint** *(specify whether from start or end of intervention)* |  | | | |
| **Results** | **Intervention** | | **Control** | |
|  | No. events | No. participants | No. events | No. participants |
|  |  |  |  |  |

Continuous outcome

|  | | **Description as stated in report/paper** | | | | | |
| --- | --- | --- | --- | --- | --- | --- | --- |
| **Comparison** | |  | | | | | |
| **Outcome** | |  | | | | | |
| **Subgroup** | |  | | | | | |
| **Timepoint** *(specify whether from start or end of intervention)* | |  | | | | | |
| **Post-intervention or change from baseline?** | |  | | | | | |
| **Results** | **Intervention** | | | | **Control** | | |
|  | Mean | | SD (or other variance) | No. participants | Mean | SD (or other variance) | No. participants |
|  |  |  |  |  |  |  |  |
