# Supplementary material for: N-acetylcysteine use among patients undergoing cardiac surgery: A systematic review and meta-analysis of randomized trials
Source: PLoS One. 2019 May 9;14(5):e0213862. doi: 10.1371/journal.pone.0213862 (PMC6508704; doi:10.1371/journal.pone.0213862)
Supplement: S4 Table — (DOCX) [file pone.0213862.s009.docx]

**Table S4.** Excluded studies with reasons.

| **Reasons of exclusions** | **References** |
| --- | --- |
| Studies that did not evaluate our pre-defined outcomes | 1. Rodrigues AJ, Evora PR, Bassetto S, Alves JL, Scorzoni AF, Origuela EA, Vicente WV. Blood cardioplegia with N-acetylcysteine may reduce coronary endothelial activation and myocardial oxidative stress. InThe heart surgery forum 2009 Jan (Vol. 12, No. 1, pp. E44-8). 2. Shafiei E, Bahtoei M, Raj P, Ostovar A, Iranpour D, Akbarzadeh S, Shahryari H, Anvaripour A, Tahmasebi R, Netticadan T, Movahed A. Effects of N-acetyl cysteine and melatonin on early reperfusion injury in patients undergoing coronary artery bypass grafting: A randomized, open-labeled, placebo-controlled trial. medicine. 2018 Jul;97(30). |
| Studies that evaluated other conditions | 1. Eshraghi A, Talasaz AH, Salamzadeh J, Salarifar M, Pourhosseini H, Nozari Y, Bahremand M, Jalali A, Boroumand MA. Evaluating the effect of intracoronary N-acetylcysteine on platelet activation markers after primary percutaneous coronary intervention in patients with ST-elevation myocardial infarction. American journal of therapeutics. 2016 Jan 1;23(1):e44-51. 2. Thayssen P, Lassen JF, Jensen SE, Hansen KN, Hansen HS, Christiansen EH, Junker A, Ravkilde J, Thuesen L, Veien KT, Jensen LO. Prevention of contrast-induced nephropathy with N-acetylcysteine or sodium bicarbonate in patients with ST-segment–myocardial infarction: A prospective, randomized, open-labeled trial. Circulation: Cardiovascular Interventions. 2014 Jan 1:CIRCINTERVENTIONS-113. 3. Erturk M, Uslu N, Gorgulu S, Akbay E, Kurtulus G, Akturk IF, Akgul O, Surgit O, Uzun F, Gul M, Isiksacan N. Does intravenous or oral high-dose N-acetylcysteine in addition to saline prevent contrast-induced nephropathy assessed by cystatin C?. Coronary artery disease. 2014 Mar 1;25(2):111-7. 4. Talasaz AH, Khalili H, Fahimi F, Jenab Y, Broumand MA, Salarifar M, Darabi F. Effects of N-acetylcysteine on the cardiac remodeling biomarkers and major adverse events following acute myocardial infarction: a randomized clinical trial. American Journal of Cardiovascular Drugs. 2014 Feb 1;14(1):51-61. 5. Berwanger O, Cavalcanti AB, Sousa AM, Buehler A, Castello-Júnior HJ, Cantarelli MJ, Mangione JA, Bergo RR, São Thiago LE, Nunes PM, da Motta PA. Acetylcysteine for the prevention of renal outcomes in patients with diabetes mellitus undergoing coronary and peripheral vascular angiography: a substudy of the acetylcysteine for contrast-induced nephropathy trial. Circulation: Cardiovascular Interventions. 2013 Jan 1:CIRCINTERVENTIONS-112. 6. Ozaydin M, Erdogan D, Yucel H, Peker O, Icli A, Akcay S, Etli M, Ceyhan BM, Sutcu R, Varol E, Dogan A. N-acetyl cysteine for the conversion of atrial fibrillation into sinus rhythm after cardiac surgery: A prospective, randomized, double-blind, placebo-controlled pilot study. International journal of cardiology. 2013 May 25;165(3):580-3. 7. Aslanger E, Uslu B, Akdeniz C, Polat N, Cizgici Y, Oflaz H. Intrarenal application of N-acetylcysteine for the prevention of contrast medium-induced nephropathy in primary angioplasty. Coronary artery disease. 2012 Jun 1;23(4):265-70. 8. Mahmoud KM, Ammar AS. Effect of N‐acetylcysteine on cardiac injury and oxidative stress after abdominal aortic aneurysm repair: A randomized controlled trial. Acta Anaesthesiologica Scandinavica. 2011 Sep;55(8):1015-21. 9. Jaffery Z, Verma A, White CJ, Grant AG, Collins TJ, Grise MA, Jenkins JS, McMullan PW, Patel RA, Reilly JP, Thornton SN. A randomized trial of intravenous N‐acetylcysteine to prevent contrast induced nephropathy in acute coronary syndromes. Catheterization and Cardiovascular Interventions. 2012 May 1;79(6):921-6. 10. Juergens CP, Winter JP, Nguyen‐Do P, Lo S, French JK, Hallani H, Fernandes C, Jepson N, Leung DY. Nephrotoxic effects of iodixanol and iopromide in patients with abnormal renal function receiving N‐acetylcysteine and hydration before coronary angiography and intervention: a randomized trial. Internal medicine journal. 2009 Jan;39(1):25-31. 11. Vallero A, Cesano G, Pozzato M, Garbo R, Minelli M, Quarello F, Formica M. Contrast nephropathy in cardiac procedures: no advantages with prophylactic use of N-acetylcysteine (NAC). Giornale italiano di nefrologia: organo ufficiale della Societa italiana di nefrologia. 2002;19(5):529-33. |
| Studies that evliuated different intervention | 1. Ozaydin M, Peker T, Akcay S, Uysal BA, Yucel H, Icli A, Erdogan D, Varol E, Dogan A, Okutan H. Addition of N‐Acetyl Cysteine to Carvedilol Decreases the Incidence of Acute Renal Injury After Cardiac Surgery. Clinical cardiology. 2014 Feb;37(2):108-14. 2. Ozaydin M, Peker O, Erdogan D, Akcay S, Yucel H, Icli A, Ceyhan BM, Sutcu R, Uysal BA, Varol E, Dogan A. Oxidative Status, Inflammation, and Postoperative Atrial Fibrillation With Metoprolol vs Carvedilol or Carvedilol Plus N‐Acetyl Cysteine Treatment. Clinical cardiology. 2014 May;37(5):300-6. 3. Ozaydin M, Icli A, Yucel H, Akcay S, Peker O, Erdogan D, Varol E, Dogan A, Okutan H. Metoprolol vs. carvedilol or carvedilol plus N-acetyl cysteine on post-operative atrial fibrillation: a randomized, double-blind, placebo-controlled study. European heart journal. 2012 Dec 11;34(8):597-604. |
| Duplicates | 1. Tossios P, Bloch W, Huebner A, Raji MR, Dodos F, Klass O, Suedkamp M, Kasper SM, Hellmich M, Mehlhorn U. N-acetylcysteine prevents reactive oxygen species–mediated myocardial stress in patients undergoing cardiac surgery: Results of a randomized, double-blind, placebo-controlled clinical trial. The Journal of thoracic and cardiovascular surgery. 2003 Nov 1;126(5):1513-20. 2. Peker O, Peker T, Erdogan D, Ozaydin M, Kapan S, Sutcu R, Ibrisim E. Effects of intravenous N-acetylcysteine on periprocedural myocardial injury after on-pump coronary artery by-pass grafting. Journal of Cardiovascular Surgery. 2008 Aug 1;49(4):527. |
