# Supplementary material for: N-acetylcysteine use among patients undergoing cardiac surgery: A systematic review and meta-analysis of randomized trials
Source: PLoS One. 2019 May 9;14(5):e0213862. doi: 10.1371/journal.pone.0213862 (PMC6508704; doi:10.1371/journal.pone.0213862)
Supplement: S5 Table — (DOCX) [file pone.0213862.s010.docx]

**Table S5.** Study characteristics related to population and setting.

| **Author**  **Year** | **Country** | **Number of randomized participants** | **Mean age per studied group** | **Male gender per group** | **Inclusion criteria** | **Exclusion criteria** | **Follow-up time (days)** |
| --- | --- | --- | --- | --- | --- | --- | --- |
| De Backer 1996^[57]^ | Belgium | 18 | I: 59 C: 63 | I: NR C: NR | NR | NR | NR |
| Eren 2003^[60]^ | Turkey | 20 | I: 61,1 C: 60,5 | I: 8 C: 7 | Primary elective CABG | Myocardial dysfunction; CAD | NR |
| Fischer 2003^[61]^ | Germany | 40 | I: 66,2 C: 66,5 | I: 12 C:19 | Primary elective CABG; urgent surgery | NR | NR |
| Vento 2003^[78]^ | Finland | 35 | I: 63,1 C: 60,2 | I: 15 C: 20 | CABG | NR | NR |
| Sucu 2004^[20]^ | Turkey | 40 | I: 66 C: 64 | I: 15 C: 14 | Primary elective CABG | EF<30% | 1 day |
| Burns 2005^[56]^ | Canada | 295 | I: 68,9 C: 69,2 | I: 116 C: 117 | Elective surgery with CPB | AKI; Creatinine level > 4.5 mg.dl^-1^; received NAC in last 5 days | NR |
| Orhan 2006^[69]^ | Turkey | 20 | I: 59,6 C: 61,8 | I: 7 C: 6 | Primary elective CABG | Inflammatory disease; use of immune suppressors; re-do operation | 2 days |

| Ristikankare 2006^[73]^ | Finland | 77 | I: 72 C: 69 | I: 28 C: 34 | Elective surgery | Kidney dysfunction; allergy to formula | NR |
| --- | --- | --- | --- | --- | --- | --- | --- |
| Koromaz 2006^[67]^ | Turkey | 30 | I: 60,2 C: 57,5 | I: 10  C: 9 | Elective CABG with CPB | EF<30% | 2 days |
| El-Hamamsy 2007^[58]^ | Canada | 100 | I: 59,8 C: 61,3 | I: 43 C: 46 | Primary elective CABG | AMI < 3 weeks; EF< 20% | 4 days |
| Haase 2007^[62]^ | Australia | 60 | I: 68,9 C: 68,3 | I: 23 C: 21 | Elective valvular /complex surgery with CPB | Allergy to formula; kidney dysfunction | NR |
| Sisillo 2008^[75]^ | Italy | 256 | I: 73 C: 72 | I: 65 C: 60 | Elective CABG | Previously using NAC; peritoneal dialysis; allergy to NAC | NR |
| Adabag 2008^[52]^ | USA | 102 | I: 70 C: 72 | I: 50 C: 52 | Elective surgery | Dialysis; kidney transplant; received contrast dye | NR |
| Barr 2008^[55]^ | USA | 38 | I: 73,8 C: 72,4 | I: 12 C: 13 | Elective cardiac surgery, urgency and emergency | Allergy to formula; kidney dysfunction | Hospital length of stay |
| Koksal 2008^[66]^ | Turkey | 30 | I: 63,4 C: 62,9 | I: 11 C: 13 | Trivascular elective surgery | Kidney dysfunction | 2 days |

| Ozaydin 2008^[70]^ | Turkey | 115 | I: 57 C: 59 | I: 47 C: 44 | Elective primary CABG or valvular surgery | NYHA III/IV; AF; LA >70mm | 2 days |
| --- | --- | --- | --- | --- | --- | --- | --- |
| Prabhu 2009^[71]^ | India | 53 | I: 54,18 C: 53,04 | NR | Primary elective CABG | EF < 0.4 | 5 days |
| Wijeysundera 2009^[79]^ | Canada | 175 | I: 74 C:73 | I: 71 C: 104 | CABG with CPB | Dialysis; use of NAC or dye contrast; allergy to NAC | 90 days |
| Karahan 2010^[63]^ | Turkey | 44 | I: 58,6 C: 56,4 | I: 12 C: 13 | Primary elective CABG | Multi-arterial disease; EF <30% | Hospital length of stay |
| Kurian 2010^[68]^ | India | 50 | I: 61,1 C: 60,1 | I: 15 C: 17 | Primary elective CABG | Antioxidants use | NR |
| Prasad 2010^[72]^ | India | 70 | I: 55,6 C: 57,77 | I: 25 C: 28 | CABG without CPB | Allergy to formula; nephrotoxic drugs; previous dialysis | NR |
| Kim 2011^[65]^ | South Korea | 48 | I: 60,8 C: 65,3 | I: 21 C: 22 | Elective primary CABG | Previous use of NAC | NR |
| Ayhan 2012^[54]^ | Turkey | 60 | I: 61,8 C: 59,9 | I: NR C: NR | Elective CABG with CPB | kidney failure; EF<35%; allergy to formula | 1 day |

| Kazemi 2013^[64]^ | Iran | 240 | I: 61,3 C: 58,2 | I: 91 C: 88 | Primary elective CABG and/or valvular surgery | LA >55mm; atrial flutter; AF; EF<25%; NYHA III/IV | 15 days |
| --- | --- | --- | --- | --- | --- | --- | --- |
| Santana-Santos 2014^[74]^ | Brazil | 70 | I: 65 c: 64 | I: 20 C: 30 | Elective CABG | Kidney dysfunction; allergy to formula | Hospital length of stay |
| Song 2015^[77]^ | South Korea | 117 | I: 68 C: 69 | I: 40 C: 43 | CABG | Kidney dysfunction; NAC within 5 pre-operative days; allergy to NAC | 2 days |
| Erdil  2016^[59]^ | Turkey | 82 | I: 58,6  C: 58,8 | I: 35  C: 34 | Elective CABG | EF <0.4 | 60 days |
| Aldemir  2016^[53]^ | Turkey | 60 | I: 71.5  C: 70.5 | I: 18  C: 22 | Elective CABG; age > 65 | Kidney dysfunction; EF<35%; allergy to NAC | NR |
| Soleimani  2018^[76]^ | Iran | 150 | I: 62.36  C: 60.70 | I: 39  C: 34 | Elective CABG | EF<30%; LA >55mm; surgery with > 4 grafts; AKI | NR |

AF: atrial fibrillation; AKI: acute kidney insufficiency; AMI: acute myocardial infarction; CABG: coronary artery bypass graft; CAD: coronary artery disease; CPB: cardiopulmonary bypass; EF: ejection fraction(cardiac); LA: left atrium; NAC: n-acetylcysteine; NR: not reported; NYHA: New York heart association.
