# Supplementary material for: N-acetylcysteine use among patients undergoing cardiac surgery: A systematic review and meta-analysis of randomized trials
Source: PLoS One. 2019 May 9;14(5):e0213862. doi: 10.1371/journal.pone.0213862 (PMC6508704; doi:10.1371/journal.pone.0213862)
Supplement: S6 Table — (DOCX) [file pone.0213862.s011.docx]

**Table S6.** Study characteristics related to intervention and control groups.

| **Author**  **year** | **Number of randomized patients in intervention and control** | **Description of intervention** | **Total dose^*^** | **Description of control** | **Measured outcomes** |
| --- | --- | --- | --- | --- | --- |
| De Backer 1996^[57]^ | I: 10  C: 8 | Intravenous + Inhaled anesthetics; Surgery with CPB: NAC 72 mg.kg^-1^.12h^-1^ pre-operative + 72 mg.kg^-1^ IV | 144 mg.kg^-1^ | SoC + Placebo | Pulmonary injury markers |
| Eren 2003^[60]^ | I: 10 C: 10 | Intravenous + Inhaled anesthetics; Surgery with CPB: NAC 100 mg.kg^-1^ IV 1h before CPB + 40 mg.kg^-1^.day^-1^ IV 24h after CPB | 140 mg.kg^-1^ | SoC + Placebo | Mortality, arrhythmia, oxidative stress markers |
| Fischer 2003^[61]^ | I: 20 C: 20 | Surgery with CPB: NAC 100 mg.kg^-1^ on CPB + 20 mg.kg^-1^.h^-1^ IV until the end of CPB | 150 mg.kg^-1^ | SoC + Placebo | Cardiac depression, mortality, AMI, oxidative stress markers |
| Vento 2003^[78]^ | I: 15 C: 20 | TIVA; Surgery with CPB: NAC 100 mg.kg^-1^ via cardioplegia | 100 mg.kg^-1^ | SoC alone | Cardiac depression, length of hospital stay, length of ICU stay, AMI, oxidative stress markers |
| Sucu 2004^[20]^ | I: 20 C: 20 | TIVA; Surgery with CPB: NAC 50 mg.kg^-1^ IV for 3 days before surgery | 150 mg.kg^-1^ | SoC + Placebo | AMI, oxidative stress markers |
| Burns 2005^[56]^ | I: 148 C: 147 | Surgery with CPB: NAC 600 mg IV intra-operative + 600 mg IV 12h post-operative + 600 mg IV 24h post-operative | 20 mg.kg^-1^ | SoC + Placebo | AKI, mortality, length of hospital stay, length of ICU stay, EVA, AMI |
| Orhan 2005^[69]^ | I: 10 C: 10 | TIVA; Surgery with CPB: NAC 50 mg.kg^-1^ IV | 50 mg.kg^-1^ | SoC alone | Mortality, length of hospital stay, length of ICU stay, arrhythmia, blood transfusion, oxidative stress markers |

| Ristikankare 2006^[73]^ | I: 38 C: 39 | Intravenous + Inhaled anesthetics; Surgery with CPB: NAC 150 mg.kg^-1^ IV after anesthesia induction + 50 mg.kg^-1^.4h^-1^ IV +100mg.kg^-1^.16h^-1^ IV | 300 mg.kg^-1^ | SoC + Placebo | AKI, mortality, ICU length of stay |
| --- | --- | --- | --- | --- | --- |
| Koromaz 2006^[67]^ | I: 15 C: 15 | Intravenous + Inhaled anesthetics; Surgery with CPB: NAC 50 mg.kg^-1^ via cardioplegia | 50 mg.kg^-1^ | SoC alone | Cardiac depression, mortality, hospital length of stay, ICU length of stay, cerebral injury, AMI, oxidative stress markers |
| El-Hamamsy 2007^[58]^ | I: 50 C: 50 | Surgery with CPB: NAC 600 mg VO 1 day before operation + 150 mg.kg^-1^ IV + 12.5 mg.kg^-1^.h^-1^ for 24h IV | 460 mg.kg^-1^ | SoC + Placebo | Cardiac depression, AKI, mortality, hospital length of stay, arrhythmia, AMI |
| Haase 2007^[62]^ | I: 30 C: 30 | Surgery with CPB: NAC 150 mg.kg^-1^ IV after anesthesia induction + 50 mg.kg^-1^.4h^-1^ IV + 100 mg.kg^-1^.20h^-1^ IV | 300 mg.kg^-1^ | SoC + Placebo | Hospital length of stay, ICU length of stay, arrhythmia, blood transfusion,  oxidative stress markers |
| Sisillo 2008^[75]^ | I: 129 C: 127 | Intravenous + Inhaled anesthetics; Surgery with and without CPB: NAC 1200 mg IV before anesthesia induction + 3 boluses of 1200 mg IV in 12h intervals | 50 mg.kg^-1^ | SoC + Placebo | Cardiac depression, AKI, mortality, AMI |
| Adabag 2008^[52]^ | I: 50 C: 52 | Surgery with and without CPB: NAC 600 mg VO twice a day total of 14 doses(3 doses before surgery + 11 doses post-operative) | 80 mg.kg^-1^ | SoC + Placebo | AKI, mortality, hospital length of stay, ICU length of stay |
| Barr 2008^[55]^ | I: 19 C: 19 | Surgery with CPB: NAC 600 mg VO twice a day 1 day before surgery + 600 mg VO 4h before surgery + 600 mg through nasogastric tube after surgery | 25 mg.kg^-1^ | SoC alone | Mortality, hospital length of stay, ICU length of stay, kidney injury markers |
| Koksal 2008^[66]^ | I: 15 C: 15 | TIVA; Surgery with CPB: NAC 4 mmol.L^-1^ (0,65 mg.mL^-1^) via cardioplegia | 10 mg.kg^-1^ | SoC alone | Cardiac depression, oxidative stress markers |

| Ozaydin 2008^[70]^ | I: 58 C: 57 | Intravenous + Inhaled anesthetics; Surgery with and without CPB: NAC 50 mg.kg^-1^ IV before anesthesia induction + 50 mg.kg^-1^.24h^-1^ IV | 100 mg.kg^-1^ | SoC + Placebo | Cardiac depression, AKI, mortality, hospital length of stay, arrhythmia, cerebral injury, blood transfusion |
| --- | --- | --- | --- | --- | --- |
| Prabhu 2009^[71]^ | I: 28 C: 25 | Intravenous + Inhaled anesthetics; Surgery with CPB: NAC 50 mg.kg^-1^ via cardioplegia | 50 mg.kg^-1^ | SoC alone | Hospital length of stay, ICU length of stay, oxidative stress markers |
| Wijeysundera 2009^[79]^ | I: 88 C: 87 | Intravenous + Inhaled anesthetics; Surgery with CPB: NAC 100 mg.kg^-1^ IV at anesthesia induction + 20 mg.kg^-1^.h^-1^ IV until 4h after CPB | 220 mg.kg^-1^ | SoC + Placebo | Mortality, blood transfusion |
| Karahan 2010^[63]^ | I: 21 C: 23 | Intravenous + Inhaled anesthetics; Surgery with CPB: NAC 50 mg.kg^-1^ via cardioplegia | 50 mg.kg^-1^ | SoC alone | Cardiac depression, hospital length of stay, ICU length of stay, AMI, oxidative stress markers |
| Kurian 2010^[68]^ | I: 25 C: 25 | Intravenous + Inhaled anesthetics; Surgery with CPB: NAC 20 mg.kg^-1^ IV before aortic clamp release | 20 mg.kg^-1^ | SoC + Placebo | AKI, mortality, AMI, oxidative stress markers |
| Prasad 2010^[72]^ | I: 35 C: 35 | Intravenous + Inhaled anesthetics; Surgery without CPB: NAC 600 mg VO twice a day 1 day before surgery + 600 mg IV at anesthesia induction + 600 mg VO twice a day until second post-operative day | 50 mg.kg^-1^ | SoC alone | AKI, hospital length of stay, ICU length of stay, blood transfusion |
| Kim 2011^[65]^ | I: 24 C: 24 | Intravenous + Inhaled anesthetics; Surgery without CPB: NAC 100 mg.kg^-1^ IV at anesthesia induction + 40 mg.kg^-1^.day^-1^ IV for 24h | 140 mg.kg^-1^ | SoC + Placebo | AKI, mortality, hospital length of stay, ICU length of stay, arrhythmia, AMI, oxidative stress markers |
| Ayhan 2012^[54]^ | I: 40 C: 20 | Intravenous + Inhaled anesthetics; Surgery with CPB: NAC 50 mg.kg^-1^ on CPB prime or 50 mg.kg^-1^ IV + 20 mg.kg^-1^ IV until the end of surgery | 50 to 130 mg.kg^-1^ | SoC + Placebo | AKI, creatinine clearance |

| Kazemi 2013^[64]^ | I: 120 C: 120 | Surgery with and without CPB: NAC 1200 mg VO.day^-1^ 48h before surgery + 1200 mg VO.day^-1^ for 72 horas after surgery | 60 mg.kg^-1^ | SoC + Placebo | Cardiac depression, AKI, mortality, hospital length of stay, ICU length of stay, arrhythmia, AMI, blood transfusion |
| --- | --- | --- | --- | --- | --- |
| Santana-Santos 2014^[74]^ | I: 35 C: 35 | Intravenous + Inhaled anesthetics; Surgery with and without CPB: NAC 150 mg.kg^-1^.2h^-1^ IV+ 50 mg.kg^-1^.6h^-1^ IV | 200 mg.kg^-1^ | SoC + Placebo | AKI, mortality, blood transfusion, kidney injury markers |
| Song 2015^[77]^ | I: 57 C: 60 | Intravenous + Inhaled anesthetics; Surgery without CPB: NAC 150 mg.kg^-1^ IV at anesthesia induction + 150 mg.kg^-1^.24h^-1^ IV | 300 mg.kg^-1^ | SoC + Placebo | Cardiac depression, AKI, hospital length of stay, ICU length of stay, blood transfusion |
| Erdil  2016^[59]^ | I: 42  C: 40 | TIVA; Surgery with CPB:  NAC 600 mg V.O. for 3 days before surgery + 300 mg via CPB prime | 17 mg.kg^-1^ | SoC + Placebo | Pulmonary function; hospital length of stay; ICU length of stay; AKI; arrhythmia |
| Aldemir  2016^[53]^ | I: 30  C: 30 | Surgery with CPB: NAC 150 mg.kg^-1^ I.V. in 15 minutes after anesthesia induction, followed by 50 mg.kg^-1^.4h^-1^ I.V. and 100 mg.kg^-1^.16h^-1^ I.V. | 300 mg.kg^-1^ | SoC + Placebo | AKI; Arrhythmia; Mortality; Hospital length of stay; ICU length of stay |
| Soleimani  2018^[76]^ | I: 72  C: 69 | TIVA; Surgery with CPB; NAC 50 mg.kg^-1.^.day^-1^ I.V. in 30 minutes for 3 days. First dose after anesthesia induction. | 150mg.kg^-1^ | SoC + Placebo | Arrhythmia; Hospital length of stay; ICU length of stay |

^*^ Total doses have been converted to express its equivalent to intravenous administration.^[42, 81]^

AKI: acute kidney insufficiency; AMI: acute myocardial infarction; CPB: cardiopulmonary bypass; EVA: encephalic vascular accident; ICU: intensive care unit; NAC: N-acetylcysteine; SoC: standard of care; TIVA: total intravenous anesthesia.
