# Supplementary material for: N-acetylcysteine use among patients undergoing cardiac surgery: A systematic review and meta-analysis of randomized trials
Source: PLoS One. 2019 May 9;14(5):e0213862. doi: 10.1371/journal.pone.0213862 (PMC6508704; doi:10.1371/journal.pone.0213862)
Supplement: S7 Table — (DOCX) [file pone.0213862.s012.docx]

**Table S7.** Risk of bias assessment.

| Author, year | **Was the randomization sequence adequately generated?** | **Was allocation adequately concealed?** | **Was there blinding of participants?** | **Was there blinding of caregivers?** | **Was there blinding of data collectors?** | **Was there blinding of statistician?** | **Was there blinding of outcome assessors?** | **Was loss to follow-up (missing outcome data) infrequent?*** | **Are reports of the study free of suggestion of selective outcome reporting?﻿** | **Was the study apparently free of other problems that could put it at a risk of bias?** |
| --- | --- | --- | --- | --- | --- | --- | --- | --- | --- | --- |
| De Backer 1996^[53]^ | Probably yes | Probably yes | Definitely yes | Probably yes | Probably yes | Probably yes | Probably yes | Probably yes | Probably yes | Probably yes |
| Eren 2003^[56]^ | Probably yes | Probably yes | Definitely yes | Probably yes | Probably yes | Probably yes | Probably yes | Probably yes | Probably yes | Probably yes |
| Fischer 2003^[57]^ | Definitely yes | Definitely yes | Definitely yes | Definitely yes | Definitely yes | Definitely yes | Definitely yes | Probably yes | Probably yes | Probably yes |
| Vento 2003^[74]^ | Probably yes | Probably no | Definitely yes | Probably no | Probably no | Probably no | Probably no | Probably yes | Probably yes | Probably yes |
| Sucu 2004^[20]^ | Probably yes | Probably no | Definitely yes | Probably no | Probably no | Probably no | Probably no | Definitely yes | Probably yes | Probably yes |
| Burns 2005^[52]^ | Definitely yes | Definitely yes | Definitely yes | Definitely yes | Definitely yes | Definitely yes | Definitely yes | Definitely yes | Probably yes | Probably yes |
| Orhan 2006^[65]^ | Probably yes | Probably yes | Definitely yes | Probably yes | Probably yes | Probably yes | Probably yes | Probably yes | Probably yes | Probably yes |
| Ristikankare 2006^[69]^ | Probably yes | Probably yes | Definitely yes | Probably yes | Probably yes | Probably yes | Probably yes | Definitely yes | Probably yes | Probably yes |
| Koromaz 2006^[63]^ | Definitely yes | Probably no | Definitely yes | Probably no | Probably no | Probably no | Probably no | Probably yes | Probably yes | Probably yes |
| El-Hamamsy 2007^[54]^ | Probably yes | Probably yes | Definitely yes | Probably yes | Probably yes | Probably yes | Probably yes | Probably yes | Probably yes | Probably yes |
| Haase 2007^[58]^ | Definitely yes | Definitely yes | Definitely yes | Definitely yes | Definitely yes | Definitely yes | Definitely yes | Definitely yes | Probably yes | Probably yes |
| Sisillo 2008^[71]^ | Definitely yes | Definitely yes | Definitely yes | Definitely yes | Definitely yes | Definitely yes | Definitely yes | Probably yes | Probably yes | Probably yes |
| Adabag 2008^[48]^ | Definitely yes | Definitely yes | Definitely yes | Definitely yes | Definitely yes | Definitely yes | Definitely yes | Definitely no | Definitely yes | Probably yes |
| Barr 2008^[51]^ | Probably yes | Definitely yes | Definitely yes | Definitely yes | Definitely yes | Definitely yes | Definitely yes | Definitely no | Probably yes | Probably yes |
| Koksal 2008^[62]^ | Definitely yes | Probably no | Definitely yes | Probably no | Probably no | Probably no | Probably no | Probably yes | Probably yes | Probably yes |
| Ozaydin 2008^[66]^ | Probably yes | Probably yes | Definitely yes | Probably yes | Probably yes | Probably yes | Probably yes | Definitely yes | Probably yes | Probably yes |
| Prabhu 2009^[67]^ | Probably yes | Probably yes | Definitely yes | Probably yes | Probably yes | Probably yes | Probably yes | Probably yes | Probably yes | Probably yes |
| Wijeysundera 2009^[75]^ | Definitely yes | Definitely yes | Definitely yes | Definitely yes | Definitely yes | Definitely yes | Definitely yes | Definitely yes | Definitely no | Probably yes |
| Karahan 2010^[59]^ | Probably yes | Probably no | Definitely yes | Probably no | Probably no | Probably no | Probably no | Definitely yes | Probably yes | Probably yes |
| Kurian 2010^[64]^ | Probably yes | Probably no | Definitely yes | Probably no | Probably no | Probably no | Probably no | Probably yes | Probably yes | Probably yes |
| Prasad 2010^[68]^ | Definitely yes | Definitely no | Probably no | Definitely no | Definitely no | Definitely no | Definitely no | Probably yes | Probably yes | Probably yes |
| Kim 2011^[61]^ | Definitely yes | Probably no | Definitely yes | Probably no | Probably no | Probably no | Probably no | Probably yes | Probably yes | Probably yes |
| Ayhan 2012^[50]^ | Definitely yes | Definitely yes | Definitely yes | Definitely yes | Definitely yes | Definitely yes | Definitely yes | Definitely yes | Probably yes | Probably yes |
| Kazemi 2013^[60]^ | Definitely yes | Definitely yes | Definitely yes | Definitely yes | Definitely yes | Definitely yes | Definitely yes | Definitely yes | Probably yes | Probably yes |
| Santana-Santos 2014^[70]^ | Definitely yes | Definitely yes | Definitely yes | Definitely yes | Definitely yes | Definitely yes | Definitely yes | Probably yes | Definitely yes | Probably yes |
| Song 2015^[73]^ | Definitely yes | Definitely yes | Definitely yes | Definitely yes | Definitely yes | Definitely yes | Definitely yes | Definitely yes | Definitely no | Probably yes |
| Erdil  2016^[55]^ | Probably yes | Probably yes | Definitely yes | Probably yes | Probably yes | Probably yes | Probably yes | Definitely yes | Probably yes | Probably yes |
| Aldemir  2016^[49]^ | Definitely yes | Definitely yes | Definitely yes | Definitely yes | Definitely yes | Definitely yes | Definitely yes | Definitely yes | Definitely yes | Definitely yes |
| Soleimani  2018^[72]^ | Definitely yes | Definitely yes | Definitely yes | Definitely yes | Definitely yes | Definitely yes | Definitely yes | Definitely yes | Definitely yes | Definitely yes |
